# MS/MS spectral tag-based annotation of non-targeted profile of plant secondary metabolites

**DOI:** 10.1111/j.1365-313X.2008.03705.x

**Published:** 2008-11-11

**Authors:** Fumio Matsuda, Keiko Yonekura-Sakakibara, Rie Niida, Takashi Kuromori, Kazuo Shinozaki, Kazuki Saito

**Affiliations:** 1 RIKEN Plant Science Center1-7-22 Suehiro-cho, Tsurumi-ku, Yokohama 230 0045, Japan; 2 Graduate School of Pharmaceutical Sciences, Chiba UniversityChiba 263 8522, Japan

**Keywords:** non-targeted metabolic profiling analysis, MS/MS spectral tag, peak annotation, liquid chromatography-mass spectrometry, secondary metabolites

## Abstract

The MS/MS spectral tag (MS2T) library-based peak annotation procedure was developed for informative non-targeted metabolic profiling analysis using LC-MS. An MS2T library of Arabidopsis metabolites was created from a set of MS/MS spectra acquired using the automatic data acquisition function of the mass spectrometer. By using this library, we obtained structural information for the detected peaks in the metabolic profile data without performing additional MS/MS analysis; this was achieved by searching for the corresponding MS2T accession in the library. In the case of metabolic profile data for Arabidopsis tissues containing more than 1000 peaks, approximately 50% of the peaks were tagged by MS2Ts, and 90 peaks were identified or tentatively annotated with metabolite information by searching the metabolite databases and manually interpreting the MS2Ts. A comparison of metabolic profiles among the Arabidopsis tissues revealed that many unknown metabolites accumulated in a tissue-specific manner, some of which were deduced to be unusual Arabidopsis metabolites based on the MS2T data. Candidate genes responsible for these biosyntheses could be predicted by projecting the results to the transcriptome data. The method was also used for metabolic phenotyping of a subset of *Ds* transposon-inserted lines of Arabidopsis, resulting in clarification of the functions of reported genes involved in glycosylation of flavonoids. Thus, non-targeted metabolic profiling analysis using MS2T annotation methods could prove to be useful for investigating novel functions of secondary metabolites in plants.

## Introduction

The objective of ‘non-targeted’ metabolic profiling analysis is to describe metabolic events in plants by determining all detectable metabolites. Of the various profiling techniques, non-targeted analysis using LC-MS is a promising tool for investigating the diversity of phytochemicals (Bottcher *et al.*, 2008; Dettmer *et al.*, 2007; Villas-Boas *et al.*, 2005); it is said to be as effective as methods employing GC-MS (Moco *et al.*, 2007b; Tikunov *et al.*, 2005). Many applications have been reported in various fields of plant sciences (Broeckling *et al.*, 2005; Farag *et al.*, 2008; Grata *et al.*, 2008; Keurentjes *et al.*, 2006; Kim *et al.*, 2007; Schliemann *et al.*, 2007), including functional genomic studies for the identification of metabolism-related genes (Messerli *et al.*, 2007; Mintz-Oron *et al.*, 2008; Schauer *et al.*, 2006). The methodology of LC-MS-based metabolic profiling has recently been improved in terms of data acquisition with the development of peak-picking software packages such as xcms (Smith *et al.*, 2006), MZmine (Katajamaa and Oresic, 2005) and MetAlign (de Vos *et al.*, 2007). The current state of the art of LC-MS metabolomics has been summarized in the experimental protocol by the Wageningen group (de Vos *et al.*, 2007) as well as in review articles (Dettmer *et al.*, 2007; Dunn, 2008).

One of the most difficult technical challenges encountered in LC-MS metabolomics is the development of an annotation strategy for the many unknown peaks (Bino *et al.*, 2004; Moco *et al.*, 2007a; de Vos *et al.*, 2007). In microarray analyses, gene expression profile data are analyzed by using various data-mining methods. In addition, functional annotations for each gene spotted on the array can be deduced from the sequence data by performing a homology search of the databases. The results are interpreted on the basis of the gene expression and annotation data, promoting further understanding of plant functions. However, metabolite information has not been fully assigned to peaks in LC-MS profile data. For example, only the peaks derived from six flavonoids, several glucosinolates and a few phenylpropanoids have been annotated in the case of the aerial tissues of intact Arabidopsis (Keurentjes *et al.*, 2006; von Roepenack-Lahaye *et al.*, 2004), while the metabolic profile data often contain more than 1000 peaks (rows). Thus, the current state of non-targeted metabolic profiling using LC-MS may be considered to be an analogy of an EST-based custom-made microarray, but one that lacks sequence information

With regard to GC-MS profiling, the peak annotation procedure has been facilitated by creation of a spectral database of authentic compound data (Wagner *et al.*, 2003), as well as improvements in the methods of processing complex profiling data (Jonsson *et al.*, 2006; Kopka, 2006; Lisec *et al.*, 2006; Tikunov *et al.*, 2005; Wiklund *et al.*, 2008). However, only a few peaks in the metabolic profile data were annotated by using a standard compound-based method in LC-MS profiling because the collection of authentic compounds of plant secondary metabolites is incomplete. Therefore, considerable efforts have been made in annotation of metabolites using tandem mass spectral data (MS/MS) (Bottcher *et al.*, 2008; Farag *et al.*, 2007; Moco *et al.*, 2006; Rochfort *et al.*, 2008; von Roepenack-Lahaye *et al.*, 2004; Suzuki *et al.*, 2007). Although the MS/MS spectra are insufficient for metabolite identification in a strict sense, they can provide an indication of putative structures of metabolites via databases and/or manual interpretation of the fragmentation pattern (Bottcher *et al.*, 2008; Rochfort *et al.*, 2008).

**Figure 1:**
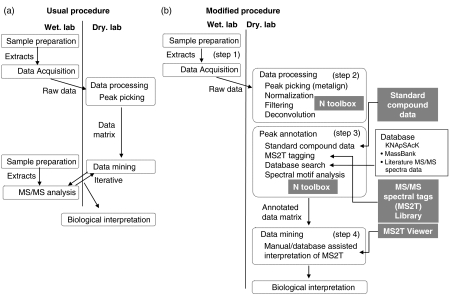
Usual (a) and modified (b) procedures for non-targeted metabolic profiling analysis using LC-MS. The new and improved steps in this study are highlighted in gray.

In non-targeted metabolic profiling analyses, MS/MS data have usually been acquired for several interesting peaks observed by data mining (Bottcher *et al.*, 2008; Cao *et al.*, 2008; Soga *et al.*, 2006; Takahashi *et al.*, 2008). Thus, additional MS/MS analyses are required when other peaks were observed by means of a different mining method (Figure 1a). This situation can be improved if the MS/MS spectra of most of the peaks in the profile data are acquired and stored in a library prior to metabolic profiling analyses (Figure 1b). A spectral library can be created from MS/MS spectra obtained using the automatic data acquisition function of the MS spectrometer in an experiment distinct from conventional metabolic profiling analyses. Once the library is created, the MS/MS spectra of the metabolite peaks observed in the profile data can be obtained from the library. This will enable deduction of the structure of the metabolites by manual and/or database-assisted interpretation of the fragmentation pattern without additional MS/MS analysis. On the basis of this information, a hypothesis can be formulated for a metabolic event in sample plants to facilitate further functional characterization of plant metabolism, as performed in microarray analyses. Identification of unusual plant constituents by interpretation of the MS2T data may reveal the existence of a pathway and the genes responsible for such biosynthesis in plants. Additionally, metabolic phenotyping of a loss-of-function mutant could provide an understanding of the function of the mutated gene.

In this study, a strategy for non-targeted metabolic profiling analysis using LC-MS with MS2T-based peak annotation was investigated by developing an MS2T library of Arabidopsis metabolites. The performance of the developed method was evaluated by analyzing the tissue specificity of the metabolites and metabolic phenotyping of *Ds* transposon-tagged mutant lines of Arabidopsis. Using this method, more than 1000 peaks were quantitatively analyzed, and approximately 50% of these peaks were tagged by MS2Ts. The MS2T-based peak annotation procedure appends metabolite information to approximately 100 of these peaks. The metabolic profile data successfully reveal not only novel aspects of tissue-specific secondary metabolism in Arabidopsis but also metabolic functions of the mutated genes by describing the metabolic events occurring in plant tissues.

## Results

### Creation of MS2T libraries

**Figure 2:**
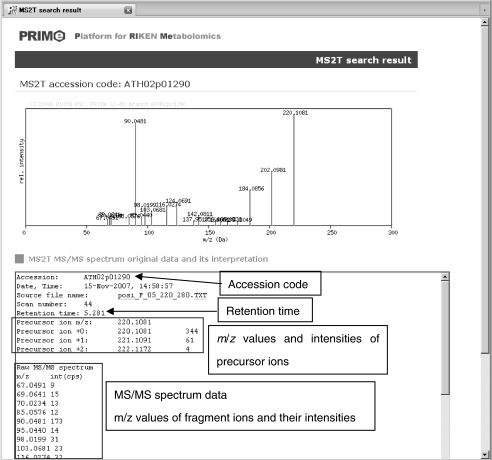
Screenshot of the MS2T viewer. The spectrum image of MS/MS data (upper panel) and other text records (retention time, precursor ion *m*/*z*, etc., in the lower text box) of the queried MS2T accession (ATH02p01290) is displayed in the web-based tool. The MS2T viewer is available on our website (http://prime.psc.riken.jp/).

In order to create MS2T libraries of Arabidopsis shoot metabolites, sample extracts derived from the shoot and inflorescence tissues of 6-week-old Arabidopsis seedlings were analyzed using liquid chromatography-quadrupole-time-of-flight/mass spectrometry (LC-Q-TOF/MS) by operating the mass spectrometer in the data-dependent acquisition mode (Hernandez *et al.*, 2006; Ishihama, 2005). MS/MS spectra of many metabolites eluted from the column were thus automatically obtained (see Experimental procedures). The MS/MS spectral data obtained using the above method are referred to as MS/MS spectral tags (MS2Ts). As the data-dependent acquisition function did not provide MS/MS spectra in the case of overlapping metabolites due to the slow data-acquisition cycle, a slower gradient curve program with half the flow rate was employed for LC methods (see Experimental procedures). Additionally, the analyses were repeated 25 times by altering the mass ranges (60 Da) used to select precursor ions in order to obtain as many MS2Ts as possible. Finally, two MS2T libraries were prepared using shoot (ATH01p, 6491 entries) and inflorescence (ATH02p, 3703 entries) tissue extracts (Table S1). Each MS2T accession was labeled in the format‘ATH02p01290′ for example; this denotes the 1290th spectrum (01290) derived from the 2nd library of *Arabidopsis thaliana* (ATH02) extracts obtained in the positive ion mode (p, positive). To visualize the MS/MS spectral data of the MS2T accessions, a web-based tool named ‘MS2T viewer’ is provided on our website (http://prime.psc.riken.jp/) (Figure 2). It should be noted that the MS2T libraries contain a large amount of data derived from artifacts or low-intensity ions, and there is redundancy due to the iterative acquisition of MS/MS spectra of the same metabolite. The quality and technical problems of the MS2T library data are discussed in Appendix S1.

### Acquisition and processing of metabolic profile data

**Figure 3:**
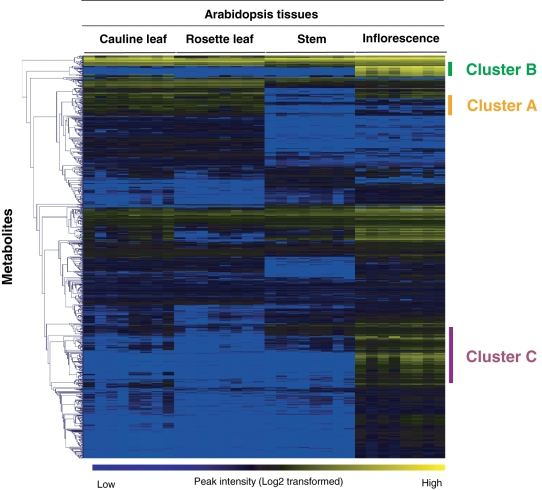
Metabolic profiles of four distinct Arabidopsis tissues. The log_2_-transformed values are represented using a heat map. Hierarchical clustering of peaks was performed for the entire metabolic profile dataset (32 columns × 1233 rows).

To compare metabolite profiles among the tissues, metabolites were extracted from the rosette leaves, cauline leaves, stems and inflorescence tissues of 6-week-old Arabidopsis seedlings (*n* = 8) and analyzed using a profiling method developed in this study (see Experimental procedures) (Figure 1b, step 1, and Figure S1). The raw chromatogram data were organized into a peak intensity table (hereafter referred to as a ‘matrix’, Table S2) using MetAlign (Moco *et al.*, 2006; de Vos *et al.*, 2007) (Figure 1b, step 2). In this matrix, peak intensity data derived from a chromatographic peak of a metabolite commonly observed among the samples (eluted at similar retention times with identical mass numbers) were recorded in a single row. Therefore, each row in the matrix consists of data including the retention time (‘Ret.’ and ‘Scan Nr’ column in Table S2), unit mass number (‘Mass’ column) and peak intensity values obtained from each sample (Table 1). The peak-picking parameters of MetAlign were selected for sensitive detection of low-intensity peaks derived from metabolites (Appendix S2). Therefore, many signals derived from data other than metabolites, such as spikes, baseline drifts and noise, were inevitably included in the matrix (data not shown), indicating that matrix filtering is essential for discarding rows containing non-metabolite peaks. In this study, the processing of the original data matrix was performed by using methods for normalization, filtering of low-intensity data, and the deconvolution of isotope peaks to produce a matrix containing fewer biased and redundant data (Figure 1b, step 2). A toolbox consisting of six tools (‘Nprefilter’, ‘Nnormalizer’, ‘Nfilter’, ‘Nisotoperemover’, ‘Nannotator’ and ‘Nmotifsearch’) has been developed to execute the corresponding data-processing steps (Appendix S2 and S3). The precision of the peak intensity was estimated to be approximately 10%, although peak height instead of peak area was used to determine peak intensity (Appendix S2); further, the drift in the retention time was restricted to within 0.1 min (data not shown). Consequently, a data matrix comprising 32 columns (samples) with 1233 rows (peaks) (Table S3) was generated from the original matrix comprising 14 946 rows (Table S2). The metabolic profiles of four tissues of Arabidopsis are shown in Figure 3. The results revealed that Arabidopsis synthesizes many phytochemicals in a tissue-specific manner.

**Table 1:** MS2T-based peak annotation results

Peak no.	Retention time (Rt) (min)	*m*/*z* (Da)	Annotation	MS2T ΔRt < 0.15 min	Compound ΔRt <0.05 min	KNApSAcK Δ*m*/*z* <5 mDa	MassBank Hit score >0.8	Literature Hit score >0.8
*Identified by cross-validation of standard compound data and database information (15 peaks)*								
204	0.888	138	Trigonelline	ATH02p00017,ATH02p00339	Trigonelline hydrochloride_CAS:6138-41-6, CAS: 535-83-1:pyridine-2-aldoximemethochloride_CAS: 51-15-0		Trigonelline	
3293	1.31	480	3-Methylsulfinyl-*n*-propylglucosinolate	ATH02p03162,ATH02p03388	3-(methylsulfinyl)propylglucosinolate_CAS: 554-88-1			
3417	1.573	494	4-Methylsulfinyl-*n*-butylglucosinolate	ATH01p01271,ATH01p01502,ATH02p03393,ATH02p03604	4-(methylsulfinyl)butylglucosinolate_CAS: 21414-41-5			
4484	1.818	613	Glutathione (oxidized form)	ATH02p04203,ATH02p04412	Glutathione (oxidized form)_CAS: 27025-41-8	C_28_H_37_O_15_: durantoside III		
99	1.911	121	[Tyramine-NH_3_]^+^	ATH02p00031			Tyramine*p*-aminobenzoate	
205	1.919	138	Tyramine		Tyramine_CAS: 51-67-2			
1350	1.928	268	Adenosine	ATH01p05470,ATH01p05473,ATH01p05679,ATH02p01278,ATH02p01281,ATH02p01572	Adenosine_CAS: 58-61-7	C_9_H_18_N_1_O_8_: miserotoxin,C_10_H_14_N_5_O_4_: adenosine,C_13_H_18_N_1_O_3_S_1_: U68204	Adenosine	
825	2.638	220	Pantothenate	ATH01p05252,ATH02p00999,ATH02p01290	Sodium d-pantothenate_CAS: 867-81-2, CAS: 79-83-4: d-Pantothenic acidhemicalcium salt_CAS: 137-08-6,CAS: 79-83-4: *trans*-zeatin_CAS:1637-39-4:		Pantothenate	
3520	3.357	505	Indol-3-ylmethylglucosinolate	ATH01p01520,ATH01p01756,ATH02p03409,ATH02p03412,ATH02p03624,ATH02p03626	4-methoxyindole-3-ylmethyl-glucosinolate_CAS: 4356-52-9		Cocarboxylase	
6090	3.5	757	Quercetin-3-*O*-α-l-rhamnopyranosyl(1,2)-β-d-glucopyranoside-7-*O*-α-l-rhamnopyranoside	ATH01p03512,ATH02p05004	Quercetin-3-*O*-α-l-rhamnopyranosyl(1,2)-β-d-glucopyranoside-7-*O*- α-l-rhamnopyranoside_CAS: 161993-01-7	C_33_H_41_O_20_: luteolin 7-rutinoside-3′-glucoside		Herbacetin-7-*O*-rha,quercetin-3′/4′-rha
5879	3.686	741	Kaempferol-3-*O*-α-l-rhamnopyranosyl(1,2)-β-d-glucopyranoside-7-*O*-α-l-rhamnopyranoside	ATH01p03327,ATH02p05006,ATH02p05009	Kaempferol-3-*O*- α-l-rhamnopyranosyl(1,2)-β-d-glucopyranoside-7-*O*- α-l-rhamnopyranoside_CAS: 162062-89-7	C_33_H_41_O_19_: apigenin7-rutinoside-4′-glucoside		Cyanidin 3-(glucoside)rhamnoside
3780	3.873	535	4-Methoxyindol-3-ylmethylglucosinolate	ATH01p01762	4-methoxyindole-3-ylmethyl-glucosinolate_CAS: 83327-21-3			
4455	3.923	611	Quercetin-3-*O*-β-glucopyranosyl-7-*O*-α-rhamnopyranoside	ATH01p02728,ATH01p02938,ATH02p04216,ATH02p04219,ATH02p04429	Quercetin-3-*O*-β-glucopyranosyl-7-*O*-α-rhamnopyranoside_CAS:18016-58-5	C_27_H_31_O_16_: isoscutellarein7-allosyl-(1→2)-glucoside,luteol, C_28_H_35_O_15_: hesperidin,neohesperidin, C_31_H_31_O_13_: 4′-*O*-methylcarthamidin 7-(2-*p*-coumaroylglucoside)		Herbacetin-7-*O*-rha,quercetin-3′/4′-rha, delphinidin3-(6′’-coumaroyl)glucoside
4285	4.211	595	Kaempferol-3-*O*-β-glucopyranosyl-7-*O*-α-rhamnopyranoside; quercetin-3,7-*O*-α-l-di-rhamnopyranoside	ATH01p02244,ATH01p02732,ATH01p02735,ATH02p04021,ATH02p04024,ATH02p04220,ATH02p04223	Kaempferol-3-*O*-β-glucopyranosyl-7-*O*-α-rhamnopyranoside_CAS:2392-95-2: Quercetin-3,7-*O*-α-l-dirhamnopyranoside_CAS:28638-13-3	C_30_H_27_O_13_: apigenin 7-(6′’-E-caffeoylglucoside);7-[[6-*O*-[3-(3,C_27_H_31_O_15_: paniculatin,apigenin 7-allosyl-(1→2)-glucoside,C_23_H_39_N_4_O_14_: didemethylallosamidin		Cyanidin 3-glucoside,cyanidin 3-galactoside,cyanidin 3-(6′’-coumaroyl)glucoside
4115	4.557	579	Kaempferol 3,7-*O*-dirhamnopyranoside	ATH01p02248,ATH01p02737,ATH01p02740,ATH02p03840,ATH02p04030	Kaempferol 3,7-*O*-dirhamnopyranoside_CAS: 482-38-2	C_28_H_35_O_13_: podorhizol β-d-glucoside,C_27_H_31_O_14_: chrysin 7-gentiobioside,7,3′,4′-trihydroxyflavone		Cyanidin 3-(glucoside)rhamnoside
*Tentatively identified by cross-validation of database information (eight peaks)*								
1649	1.446	308	Glutathione (reduced form)	ATH01p05951,ATH01p06241,ATH02p01565,ATH02p01876		C_10_H_18_N_3_O_6_S_1_: l-glutathione,C_14_H_14_N_1_O_7_: lycoricidinol		Glutathione (reduced form)
465	1.835	182	Tyrosine	ATH01p04635,ATH01p04938			Tyr	
350	2.486	166	Phenylalanine	ATH01p03885,ATH01p04646,ATH02p00363,ATH02p00684,ATH02p00687		C_9_H_12_N_1_O_2_:l-phenylalanine	Phe *N*-acetylphenylalanine,Bestatin	
666	3.145	205	Tryptophan	ATH01p04959,ATH01p05257,ATH02p01007		C_11_H_13_N_2_O_2_:l-tryptophan,vasicinol, 11-oxocytisine,C_7_H_13_N_2_O_5_: trehalamine,C_12_H_13_O_3_: 3-butylidene-7-hydroxyphthalide,C_9_H_17_O_3_S: 2-oxo-8-methylthiooctanoic acid	Trp	
3000	4.245	449	Quercetin-3,7-*O*-α-l-di-rhamnopyranoside(fragment)	ATH01p00839,ATH01p01074,ATH02p02980,ATH02p02983,ATH02p03204,ATH02p03207	Luteolin-8-C-glucoside_CAS:28608-75-5	C_21_H_21_O_11_: fisetin 8-C-glucosideC_25_H_21_O_8_: artonin P, C_18_H_25_O_13_:aralidioside		
2849	4.557	433	Kaempferol 3,7-*O*-dirhamnopyranoside(fragment)	ATH01p00842,ATH01p00845,ATH01p01079,ATH02p02985,ATH02p02988,ATH02p03209	Apigenin 8-C-glucoside_CAS: 3681-93-4	C_25_H_21_O_7_: calomelanol G; 3,4,7,8-tetrahydro-5-hydroxy-4-(4-hy,C_24_H_33_O_5_S_1_: (*S*)-furanopetasitin		
516	3.137	188	[Trp-NH_3_]^+^	ATH01p04655,ATH01p04960,ATH02p00374,ATH02p00695		C_11_H_10_N_1_O_2_: indole-3-acrylic acid	Trp	
3287	5.073	479	Isorhamnetin-3-*O*-glucoside		Isorhamnetin-3-*O*-glucoside_CAS: 5041-82-7			
*Peaks of flavonol glycosides tentatively annotated by motif analysis (24 peaks)*								
5708	4.557	725	Kaempferol-triRha	ATH02p05021				
4010	4.228	565	Kaempferol(tetrahydroxy flavone)-Rha-pentoside	ATH01p02012,ATH02p03835				Cyanidin 3-(glucoside)rhamnoside
4284	3.83	595	Kaempferol (tetrahydroxy flavone)-Hex-Rha	ATH01p02239,ATH01p02726,ATH01p02729,ATH02p04018		C_34_H_27_O_10_: agathisflavonetetramethyl ether,cupressuflavone,C_27_H_31_O_15_:paniculatin, apigenin7-allosyl-(1→2)-glucoside		Cyanidin 3-glucoside,cyanidin 3-galactoside,kaempferol-7-*O*-neohesperidoside
5883	4.312	741	Kaempferol (tetrahydroxy flavone)-Hex-diRha	ATH01p03336,ATH01p03339,ATH02p05017		C_44_H_37_O_11_:guibourtinidol-(4α→ 2)-3,5,4′-trihydroxystilben		Cyanidin 3-(glucoside)rhamnoside, cyanidin3-(6′’-coumaroyl)glucoside
6091	4.05	757	Kaempferol (tetrahydroxy flavone)-diHex-Rha; quercetin-Hex-diRha	ATH01p03518,ATH02p05012		C_44_H_37_O_12_: guibourtinidol-(4α→2)-3,5,3′,4′-tetrahydroxys,		Cyanidin 3-(glucoside)rhamnoside
4456	4.312	611	Kaempferol (tetrahydroxy flavone)-diHex	ATH01p02734,ATH01p02943,ATH02p04222,ATH02p04225,ATH02p04434,ATH02p04436		C_28_H_35_O_15_: hesperidin, neohesperidin,4,2′,4′-trihydroxy-6′-m, C_31_H_31_O_13_:4′-*O*-methylcarthamidin 7-(2-*p*-coumaroylglucoside), C_34_H_31_N_2_O_9_: atalanine,C_27_H_31_O_16_: isoscutellarein 7-allosyl-(1→2)-glucoside, luteol, C_30_H_27_O_14_:prodelphinidin B4		Cyanidin 3-glucoside, cyanidin3-galactoside, cyanidin3-sophoroside, cyanidin3-diglucoside, cyanidin3-laminaribiose
3001	4.312	449	Kaempferol (tetrahydroxy flavone)-diHex (fragment)	ATH01p00839,ATH01p01074,ATH02p02983,ATH02p03204,ATH02p03207		C_21_H_21_O_11_: fisetin 8-C-glucosideC_25_H_21_O_8_: artonin P,C_18_H_25_O_13_: aralidioside		
2848	4.211	433	Kaempferol (tetrahydroxy flavone)-3-*O*-β-glucopyranosyl-7-*O*-α-rhamnopyranoside (fragment)	ATH01p00837,ATH01p01072,ATH02p02979,ATH02p02982,ATH02p03203,ATH02p03206		C_25_H_21_O_7_: calomelanol G,C_21_H_21_O_10_: apigenin 7-*O*-glucoside,isovitexin, C_26_H_25_O_6_: artocommunol CA;(+)-6-hydroxy-11-methoxy-3,3-dimet		
4283	3.686	595	Kaempferol (tetrahydroxy flavone)-3-*O*-α-l-rhamnopyranosyl(1,2)-β-d-glucopyranoside-7-*O*-α-l-rhamnopyranoside (fragment)	ATH01p02235,ATH01p02726,ATH02p04014,ATH02p04212		C_27_H_31_O_15_(3): paniculatin, apigenin7-allosyl-(1→2)-glucoside,C_34_H_27_O_10_: agathisflavonetetramethyl ether, cupressuflavone,		Cyanidin 3-(glucoside)rhamnoside, cyanidin3-(6′’-coumaroyl)glucoside
2847	3.686	433	Kaempferol (tetrahydroxy flavone)-3-*O*-α-l-rhamnopyranosyl(1,2)-β-d-glucopyranoside-7-*O*-α-l-rhamnopyranoside (fragment)	ATH01p00829,ATH01p01063,ATH02p02971,ATH02p03196		C_21_H_21_O_10_: apigenin 7-*O*-glucoside,isovitexin, C_24_H_17_O_8_: kaempferol 3-*p*-coumarate		
5200	4.27	681	Kaempferl (tetrahydroxy flavone)-Hex-Rha-malonyl	ATH01p03162,ATH02p04625,ATH02p04818		C_34_H_33_O_15_: okanin 4′-(2′’,4′’-diacetyl-6′’-*p*-coumarylglucoside, C_30_H_33_O_18_: luteolin7-(6′’-malonylneohesperidoside), kaempfer,		
4127	4.033	581	Quercetin (pentahydroxy flavone)-Rha-pentoside	ATH01p02241,ATH01p02730,ATH02p04020,ATH02p04217		C_33_H_25_O_10_: sciadopitysin, 7,7′’,4′’’-tri-*O*-methylagathisflavo		Herbacetin-7-*O*-rha,quercetin-3′/4′-rha,herbacetin-7-*O*-rha-8-*O*-glu
4454	3.517	611	Quercetin (pentahydroxy flavone)-Hex-Rha	ATH01p02723,ATH01p02932,ATH02p04210,ATH02p04420,ATH02p04423		C_31_H_31_O_13_: 4′-*O*-methylcarthamidin 7-(2-*p*-coumaroylglucoside)		Delphinidin 3-(6′’-coumaroyl)glucoside,delphinidin 3-rutinoside,delphinidin 3-glucoside,rutin, delphinidin3-galactoside
4661	4.042	627	Quercetin (pentahydroxy flavone)-diHex	ATH01p02939,ATH02p04218,ATH02p04430		C_27_H_31_O_17_: 6-hydroxyluteolin 7-sophoroside,6-hydroxyluteolin, C_35_H_31_O_11_: kuwanon L,C_30_H_27_O_15_: 6-hydroxykaempferol 7-(6′’-(E)-caffeylglucoside)		Delphinidin 3-glucoside,delphinidin 3-galactoside
3155	4.042	465	Quercetin (pentahydroxyflavone)-diHex (fragment)	ATH01p01069,ATH01p01301,ATH02p03202,ATH02p03419		C_21_H_21_O_12_: gossypetin 8-rhamnoside,C_18_H_25_O_12_S_1_: paederosidic acid,		
2999	3.94	449	Quercetin (pentahydroxy flavone)-3-*O*-β-glucopyranosyl-7-*O*-α-rhamnopyranoside (fragment)	ATH01p00833,ATH01p01067,ATH02p02977,ATH02p03201		C_21_H_21_O_11_: fisetin 8-C-glucoside;8-C-glucosylfisetin, isoorie,C_25_H_21_O_8_: 8,9-dihydro-6,11-dihydroxy-3,3-dimethyl-		
2998	3.517	449	Quercetin (pentahydroxy flavone)-3-*O*-α-l-rhamnopyranosyl(1,2)-β-d-glucopyranoside-7-*O*-α-l-rhamnopyranoside	ATH01p00826,ATH01p01060,ATH02p02969		C_21_H_21_O_11_: fisetin 8-C-glucoside;8-C-glucosylfisetin, isoorie,		
4286	4.371	595	Isorhamnetin (tetrahydroxymethoxyflavone)-Rha-pentoside	ATH01p02735,ATH02p04024,ATH02p04027,ATH02p04223		C_27_H_31_O_15_: paniculatin,apigenin 7-allosyl-(1→2)-glucoside, C_34_H_27_O_10_:agathisflavone tetramethylether		
5545	4.625	711	Isorhamnetin(tetrahydroxymethoxyflavone)-Hex-Rha-malonyl	ATH02p04819				
4635	3.915	625	Isorhamnetin(tetrahydroxymethoxyflavone)-Hex-Rha	ATH02p04215,ATH02p04427		C_25_H_33_N_6_O_13_: nikkomycin		Petunidin 3-glucoside,petunidin 3-galactoside
4637	4.27	625	Isorhamnetin(tetrahydroxymethoxyflavone)-Hex-Rha	ATH02p04221,ATH02p04224,ATH02p04435		C_27_H_29_O_17_: luteolin 7-glucuronide-3′-glucoside		Petunidin 3-(6′’-coumaroyl)glucoside
3139	4.261	463	Isorhamnetin(tetrahydroxymethoxyflavone)-Hex-Rha (fragment)	ATH01p01305,ATH02p03205,ATH02p03423				Brevifoliol
4432	4.625	609	Isorhamnetin(tetrahydroxymethoxyflavone)-diRha	ATH01p02738,ATH01p02947,ATH02p04029,ATH02p04032,ATH02p04228		C_28_H_33_O_15_: physcion 8-gentiobioside,luteolin 3′-methyl ether, C_35_H_29_O_10_:olivieriflavone		
3140	4.625	463	Isorhamnetin(tetrahydroxymethoxyflavone)-diRha (fragment)	ATH02p03210,ATH02p03428,ATH02p03431		C_25_H_19_O_9_: sapurimycin		Brevifoliol
*Peaks of sinapoylmalate tentatively annotated by the motif analysis (four peaks)*								
691	4.845	207	Sinapoymalate (fragment)	ATH01p04987,ATH01p05285,ATH02p00722,ATH02p01034,ATH02p01037		C_10_H_11_N_2_O_1_S_1_: 3-indolylmethylthiohydroximate,C8H15O2S2: (*R*)-lipoic acid		
692	4.921	207	Sinapoymalate (isomer, fragment)	ATH01p04991,ATH01p05285,ATH01p05288,ATH02p00727,ATH02p01034, ATH02p01037		C_10_H_11_N_2_O_1_S_1_: 3-indolylmethylthiohydroximate,C_11_H_11_O_4_: lathodoratin, scoparone, C_8_H_15_O_2_S_2_:(*R*)-lipoic acid		
5832	4.921	737	Sinapoymalate (isomer, adduct)	ATH01p03345, ATH02p05025				
5831	4.837	737	Sinapoymalate (adduct)	ATH01p03345, ATH02p05025				
*Peaks of glucosinolates tentatively annotated by the motif analysis (26 peaks)*								
2199	0.981	358	4-Methylsulfinyl-*n*-butylglucosinolate (fragment)	ATH01p00006, ATH01p00268,ATH02p02175, ATH02p02178,ATH02p02438		C_20_H_12_N_3_O_4_: BE 13793C		
602	0.981	196	4-Methylsulfinyl-*n*-butylglucosinolate (fragment)	ATH01p04622, ATH02p00659,ATH02p00662, ATH02p00971,ATH02p00974				
2200	1.564	358	4-Methylsulfinyl-*n*-butylglucosinolate (fragment)	ATH01p00011, ATH01p00278,ATH02p02185		C_20_H_12_N_3_O_4_: BE 13793C	Tyrosine methyl ester,glucosaminate	
603	1.564	196	4-Methylsulfinyl-*n*-butylglucosinolate (fragment)	ATH01p04630, ATH02p00668,ATH02p00980				
2310	1.962	372	5-Methylsulfinyl-*n*-pentylglucosinolate (fragment)	ATH01p00015				2α-Scetoxy-2′β-deacetylaustrospicatine
727	1.962	210	5-Methylsulfinyl-*n*-pentylglucosinolate (fragment)					
3541	1.962	508	5-Methylsulfinyl-*n*-pentylglucosinolate_1	ATH01p01504, ATH01p01741,ATH01p01743, ATH02p03397,ATH02p03610		C_10_H_16_N_5_O_13_P_2_S_1_:3′-phosphoadenosine5′-phosphosulfate	Loperamide,albendazole,*N*6-methyl-2′-deoxyadenosine	
3669	2.325	522	6-Methylsulfinyl-*n*-hexylglucosinolate	ATH01p01508, ATH01p01746,ATH02p03614, ATH02p03812				
2572	2.799	400	7-Methylsulfinyl-*n*-heptylglucosinolate(fragment)	ATH01p00563, ATH01p00566,ATH01p00815, ATH02p02722,ATH02p02959		C_20_H_22_N_3_O_6_: pelagiomicin A	Cloquintocet-mexyl,ketamine	
1102	2.799	238	7-Methylsulfinyl-*n*-heptylglucosinolate(fragment)	ATH02p01000, ATH02p01292				
3785	2.799	536	7-Methylsulfinyl-*n*-heptylglucosinolate	ATH01p01747, ATH01p01991,ATH02p03619, ATH02p03621,ATH02p03816, ATH02p03819			Simeconazole,triadimefon	
3275	3.044	478	4-Methylthio-*n*-butylglucosinolate	ATH01p01287, ATH01p01290,ATH01p01515, ATH02p03188,ATH02p03406				
2057	3.052	342	4-Methylthio-*n*-butylglucosinolate(fragment)	ATH01p00029, ATH01p06265,ATH02p02207		C_17_H_16_N_3_O_5_: pelagiomicin C		
3903	3.314	550	8-Methylsulfinyl-*n*-octylglucosinolate	ATH01p02001, ATH01p02230,ATH01p02233, ATH02p03821,ATH02p04007, ATH02p04010				
2690	3.323	414	8-Methylsulfinyl-*n*-octylglucosinolate(fragment)	ATH01p00573, ATH01p00825,ATH02p02728, ATH02p02966		C_17_H_24_N_3_O_9_: SB 219383,C_22_H_24_N_1_O_7_: α-narcotine,synerazol		
1230	3.323	252	8-Methylsulfinyl-*n*-octylglucosinolate(fragment)	ATH01p05491, ATH01p05699,ATH02p01299, ATH02p01302,ATH02p01595		C_10_H_14_N_5_O_3_: cordycepin,oxetanocin		
2284	3.348	369	Indol-3-ylmethylglucosinolate (fragment)	ATH01p00036, ATH02p02211,ATH02p02472		C_20_H_17_O_7_: averufin, velloquercetin,malaccol C_16_H_21_N_2_O_6_S_1_(1): 3-indolylmethyldesulfoglucosinolate		
685	3.348	207	Indol-3-ylmethylglucosinolate (fragment)	ATH01p04963, ATH01p05260,ATH02p00699		C_11_H_11_O_4_: lathodoratin, scoparone,C_10_H_11_N_2_O_1_S_1_: 3-indolylmethylthiohydroximate, C_8_H_15_O_2_S_2_: (*R*)-lipoic acid		
2557	3.873	399	4-Methoxyindol-3-ylmethylglucosinolate(fragment)	ATH01p00310, ATH01p00581				
1091	3.881	237	4-Methoxyindol-3-ylmethylglucosinolate(fragment)	ATH01p05268, ATH01p05499		C_14_H_9_N_2_O_2_: 11-hydroxycanthin-6-one,C_12_H_13_O_5_: 5,6,7-trimethoxycoumarin,orthosporin, NSC 118343		
2558	4.464	399	1-Methoxyindol-3-ylmethylglucosinolate(fragment)	ATH01p00320, ATH01p00590				
859	5.251	222	7-Methylthio-*n*-heptylglucosinolate (fragment)	ATH01p05291				
3649	5.251	520	7-Methylthio-*n*-heptylglucosinolate	ATH01p01544, ATH01p01780				
1078	5.987	236	8-Methylthio-*n*-octylglucosinolate (fragment)	ATH01p05300, ATH01p05525				
2549	5.987	398	8-Methylthio-*n*-octylglucosinolate (fragment)	ATH01p00342				
3774	5.987	534	8-Methylthio-*n*-octylglucosinolate	ATH01p01786, ATH01p02036				
*Peaks of hydroxycinnamoylspermidines tentatively annotated by the motif analysis (16 peaks)*								
6168	5.877	764	Spermidine-trisinapyl	ATH02p05211				
5681	5.53	722	Spermidine-trihydroxyferuloyl	ATH01p03349, ATH02p04829,ATH02p05032				
3184	4.6	468	Spermidine-*p*-coumaroyl-feruloyl	ATH01p01080, ATH01p01310,ATH02p03212, ATH02p03430		C_27_H_34_N_1_O_6_: (+)-pyripyropene G		
3869	5.877	544	Spermidine-hydroxyferuloyl-sinapyl	ATH01p02033, ATH02p03657,ATH02p03660, ATH02p03854,ATH02p03856				
6022	6.232	750	Spermidine-hydroxyferuloyl-disinapyl	ATH01p03534, ATH02p05042				
3711	4.557	528	Spermidine-feruloyl-sinapyl	ATH02p03642, ATH02p03842				
3589	4.388	514	Spermidine-feruloyl-hydroxyferuloyl	ATH02p03425, ATH02p03636				
3956	4.676	558	Spermidine-disinapyl	ATH01p02018, ATH02p03841				
4341	3.83	600	Spermidine-di-*p*-coumaroyl-caffeoyl	ATH02p04015, ATH02p04214				β-d-Glucopyranoside,(2E)-3-(4-methoxyphenyl)-2-propenyl 6-*O*-α-l-arabinopyranosyl-
2905	4.659	438	Spermidine-di-*p*-coumaroyl	ATH01p00843, ATH01p00846,ATH01p01078, ATH01p01081,ATH02p02987, ATH02p02991,ATH02p03211, ATH02p03214		C_25_H_32_N_3_O_4_: lunarine,C_12_H_24_N_1_O_10_S_3_: 4-methylsulfinylbutyl glucosinolate,C_26_H_32_N_1_O_5_: decaline		
5827	5.902	736	Spermidine-dihydroxyferuloyl-sinapyl	ATH01p03353, ATH02p05036,ATH02p05038				
3733	5.53	530	Spermidine-dihydroxyferuloyl	ATH02p03850				
5301	6.325	690	Spermidine-diferuloyl-hydroxyferuloyl	ATH02p04640, ATH02p04838				
3454	4.87	498	Spermidine-diferuloyl	ATH01p01312, ATH01p01539,ATH02p03432, ATH02p03644				
5493	5.911	706	Spermidine-caffeoyl-hydroxyferuloyl-sinapyl	ATH01p03354, ATH02p04834,ATH02p05037				
7815	5.386	898	Spermidine-caffeoyl-dihydroxyferuloyl-sinapyl	ATH02p05422				

### Annotation of peaks using standard compounds

For annotation of peaks in the matrix, the retention time (min) and mass number (*m*/*z*) of commercially available standard compounds in addition to those of the authentic Arabidopsis standards (280 compounds in total) were acquired by the same profiling analysis method (Table S4). For each peak in the matrix, we searched standard compound data for a compound with an identical *m*/*z* value (unit mass data) that eluted at a similar retention time (within 0.05 min) (Figure 1b, step 3). Thirty-five matched pairs were obtained, and the annotation information is described under the heading ‘Compound’ in Table S3.

### MS2T-based peak annotation

As MS2T data contain information about the retention time and *m*/*z* value of the precursor ion (Figure 2), the peaks in the matrix with identical *m*/*z* values that eluted at similar retention times (within 0.15 min) could be tagged with MS2T accessions (Figure 1b, step 3). A total of 614 peaks in the matrix were tagged by at least one MS2T. The results are listed in the ‘MS2T’ column in Table S3. The MS2T data tagged to each peak in the matrix were queried in three databases, including KNApSAcK (Oikawa *et al.*, 2006; Shinbo *et al.*, 2006), MassBank (Taguchi *et al.*, 2007) and our in-house database of MS/MS spectral data taken from the literature (Figure 1b, step 3). Putative structural information was obtained for peaks 207, 69 and 41 in the matrix, as described in the ‘KNApSAcK’, ‘MassBank’ and ‘Literature’ columns of the matrix, respectively (Table S3). However, these tentative annotations are likely to include many false positives. Thus, the annotation information was cross-validated among the annotation methods to find plausible annotations. For example, the 825th peak (*m*/*z* 220; retention time 2.64 min) in the matrix was annotated as the protonated molecule [M + H]^+^ of d-pantothenate based on standard compounds and the MS2T data (ATH02p01290, Figure 2), which is essentially identical to the result using the MassBank MS/MS spectrum data (KO003696, pantothenate) with a hit score of 0.950. A total of 15 and eight peaks were identified and tentatively annotated based on the standard compound and MS2T data.

### Detection of structurally related metabolites by a spectral motif search

**Figure 4:**
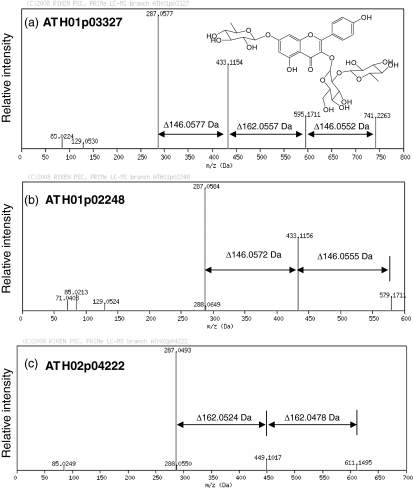
MS/MS spectra of the MS2Ts tagged to (a) the 5879th peak (ATH01p03327, kaempferol-3-*O*-rhamnosyl(1,2)-glucoside-7-*O*-rhamnoside), (b) the 4115th peak (ATH01p02248, kaempferol-3,7-*O*-dirhamnoside), and (c) the 4465th peak (ATH02p0422, kaemferol dihexoside). The deduced neutral losses of hexose (Δ162.0528 Da) and rhamnose (Δ146.0579 Da) are indicated in the spectra.

It is well recognized that plants often contain a series of metabolites with similar structures. For example, it is expected that Arabidopsis will produce dozens of flavonols with various glycosylation patterns. The MS/MS spectra of two kaempferol glycosides identified above [ATH01p03327 of the 5879th peak (kaempferol-3-*O*-rhamnosyl(1,2)-glucoside-7-*O*-rhamnoside, Figure 4a) and ATH01p02248 of the 4115th peak (kaempferol-3,7-*O*-dirhamnoside, Figure 4b)] indicated that occurrence of the fragment ion of the kaempferol aglycon moiety (C_15_H_11_O_6_; *m*/*z* 287.0556) together with the neutral loss of glucose (C_6_H_10_O_5_; *m*/*z* 162.0528) and rhamnose (C_6_H_10_O_4_; *m*/*z* 146.0579) is a common spectral ‘motif’ in these MS/MS spectra. These results suggest that the peaks of structurally related metabolites can be extracted from the matrix by identifying MS2Ts containing the same spectral ‘motif.’ Here, the motif of kaempferol glycosides was defined by regular expression of the MS/MS spectral data as follows: frg (C15H11O6) && (nl (C6H10O5) || nl (C6H10O4)).

The above formula indicates the spectral motif containing the fragment ion of kaempferol aglycon (tetrahydroxy flavone, in the strict sense) [frg (C15H11O6)] with the neutral loss of hexose [nl (C6H10O5)] or deoxyhexose [nl(C6H10O4)]. The formula was queried against MS2T libraries to search for peaks derived from structurally related metabolites using an ‘Nmotifsearch’ program written in Perl/Tk. Consequently, 10 additional peaks of kaempferol (tetrahydroxy flavone) glycosides or their fragment ions were tentatively determined (Table 1). Among them, kaempferol (tetrahydroxy flavone) dihexose (ATH02p04222 of the 4465th peak; Figure 4c) has not been reported previously as an Arabidopsis metabolite. Using this procedure, molecular-related or fragment ions of flavonol and glucosinolate derivatives were assigned to 24 and 26 peaks in total, respectively. Thus a total of 95 peaks derived from 44 metabolites were identified or tentatively annotated by this procedure (Table 1 and Table S5).

### Inter-tissue comparison of metabolite profiles in the aerial parts of Arabidopsis

**Figure 5:**
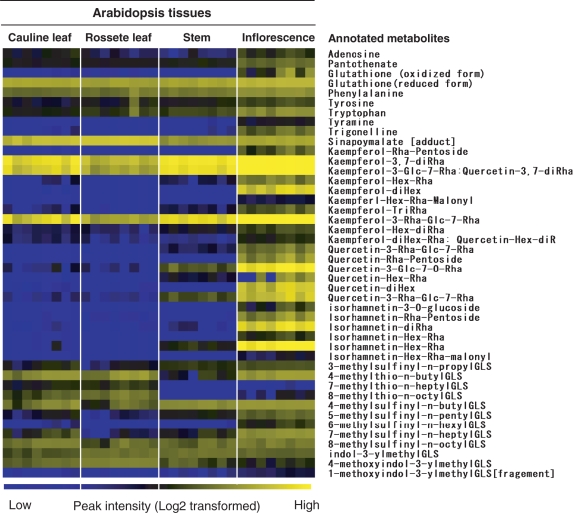
Inter-tissue comparison of the levels of 44 identified metabolites. The log_2_-transformed intensity values are represented using a heat map.

It has been suggested that plants produce various types of phytochemicals in a tissue-specific manner. However, the overall difference in metabolic profiles among the tissues has not been thoroughly investigated. To understand the tissue-specific metabolism in Arabidopsis, the metabolic profiles of the 44 metabolites identified or tentatively deduced by the MS2T annotation method were compared among the tissues (Figure 5). The metabolic profiles in cauline leaves, rosette leaves and stem tissues were similar to each other, except for a significant decrease in the levels of methylthioglucosinolates in the stem (Figure 5). This downregulation can partly be explained by upregulation of the *S*-oxygenating enzyme gene (At1g65860) that catalyzes the conversion of methylthioglucosinolates to the corresponding methylsulfinylglucosinolates in stem tissue (Hansen *et al.*, 2007) (Figure S2a). In contrast, the profiles in the inflorescence tissues changed drastically due to accumulation of tyramine, quercetin and isorhamnetin glycosides as well as methylsulfinylglucosinolates (Brown *et al.*, 2003). This coincided with the active expression of these biosynthesis-related genes in the flower, such as the *OMT1* gene (At5g54160), which has a dual function in methylation of quercetin aglycon to isorhamnetin (Tohge *et al.*, 2007) in addition to lignin biosynthesis. Comparison of the gene expression data of *OMT1* with the metabolic profile data revealed that the methylation of quercetin to isorhamnetin in the stem was less than that in the inflorescence tissues, while *OMT1* was also highly expressed in stem, probably for active lignin biosynthesis (Figure S2b). These results suggest that flavonol glycosides and lignin are specifically biosynthesized in stem tissues.

**Figure 6:**
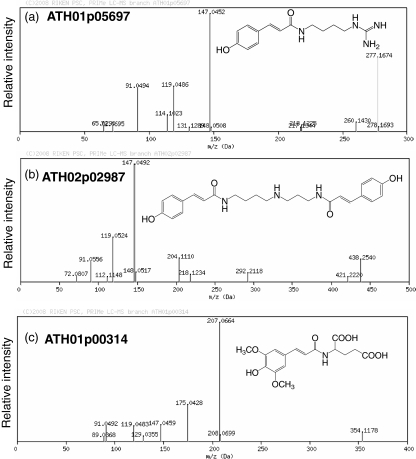
MS/MS spectra of (a) the 1408th peak (ATH01p05697, *p*-coumaroylagmatine, putative), (b) the 2905th peak (ATH02p02987, di-*p*-coumaroylspermidine, putative) and (c) the 2156th peak (ATH01p00314, sinapoylglutamate, putative). Tentatively deduced structures are also shown.

To investigate further tissue-specific secondary metabolism in Arabidopsis, the metabolic profile data shown in Figure 3 were characterized to identify novel Arabidopsis metabolites by interpreting the MS2T data (Figure 1b, step 4). Despite their morphological differences, the metabolite profiles of rosette and cauline leaves are very similar to each other, suggesting that these leaves have similar metabolic functions. However, one peak (peak number 1408, *m*/*z* 277) that eluted at 3.23 min was specifically observed in the case of rosette leaf samples (Table 2). The metabolite responsible for this peak was determined to be *p*-coumaroylagmatine by manual interpretation of MS2T data (ATH01p05697, Figure 6a and Figure S3a), and this was confirmed by data from the literature (von Ropenack *et al.*, 1998). The most remarkable metabolic phenotype was observed in the inflorescence tissues, where there was accumulation of several metabolites (clusters B and C in Figure 3). Of the peaks in cluster B, the intensities of five peaks drastically increased in an inflorescence tissue-specific manner (Table 2). Interpretation of the MS2T data tagged to these peaks revealed that the five metabolites corresponding to these peaks were di- or trihydroxycinnamic acid amides of spermidines such as di-*p*-coumaroylspermidine (ATH02p02987, Figure 6b and Figure S3b); this was supported by literature data (Bottcher *et al.*, 2008; Youhnovski *et al.*, 2001). A spectral motif search [query text: nl(C3H7N)] revealed that an additional 11 structurally related metabolites accumulated during the inflorescence process (Table 1). Among them, di-sinapoylspermidine has recently been reported as a seed metabolite of Arabidopsis (Bottcher *et al.*, 2008; Meissner *et al.*, 2008). Another inflorescence-specific metabolite (peak number 2156, retention time 3.957, *m*/*z* 344) was tentatively identified from cluster C in Figure 3 as sinapoylglutamate by interpretating the MS2T data (ATH01p00314, Figure 6c and Figure S3c). The identification of *p*-coumaroylagmatine, di-*p*-coumaroylspermidine and sinapoylglutamate in Arabidopsis tissues suggests that Arabidopsis has many unknown metabolic functions that remain to be uncovered. In addition, it should be noted that the peak annotations given here were obtained by referring MS2Ts without additional MS/MS data acquisition work (Figure 1b).

**Table 2:** Deduced annotation, MS2T data and relative peak intensity of the inflorescence tissue-specific metabolites

						Relative intensity (internal standard = 1.0)			
Peak no.	Retention time (min)	Mass (*m*/*z*)	Tentative annotation	MS2T code (representative)	MS2T data *m*/*z* (relative intensity)	Inflorescence	Cauline leaf	Rosette leaf	Stem
1408	3.23	277	*p*-Coumaroylagmatine, putative	ATH01p05697	91.0494 (37), 114.1023 (14),119.0486 (48), 147.0452 (100),218.1225 (6), 260.1430 (8)	0.004 ± 0.002	0.003 ± 0.001	0.065 ± 0.037	0.003 ± 0.001
2156	3.957	344	Sinapoylglutamate, putative	ATH01p00314	91.0492 (13), 119.0483 (13),147.0459 (14), 175.0428 (30),207.0664 (100)	0.045 ± 0.008	0.010 ± 0.005	0.004 ± 0.001	0.006 ± 0.003
2905	4.66	438	Di-*p*-coumaroylspermidine,putative	ATH02p02987	91.0556 (18), 119.0524 (41),147.0492(100), 204.1110 (19),292.2118 (8), 438.2540 (20)	0.880 ± 0.292	0.003 ± 0.001	0.003 ± 0.000	0.003 ± 0.001
3453	4.69	498	Di-feruloylspermidine, putative	ATH02p03429	72.0766 (5), 117.0284 (5),145.0239 (17), 177.0498 (45),234.1055 (24), 305.1812 (8),322.2060 (29), 498.2472 (100)	0.669 ± 0.320	0.003 ± 0.001	0.003 ± 0.000	0.003 ± 0.001
5681	5.53	722	Tri-hydroxyferuroylspermidine,putative	ATH02p04827	193.0454 (46), 250.0949 (50),530.2488 (100), 722.2715 (65)	0.877 ± 0.593	0.014 ± 0.012	0.003 ± 0.000	2.974 ± 0.623
5827	5.90	736	Di-hydroxyferuroyl-sinapoylspermidine, putative	ATH02p05036	161.0204 (6), 175.0361 (8),193.0451 (15), 207.0619 (14),250.1024 (23), 321.1768 (7),338.2016 (9), 352.2197 (9),526.2460 (18), 544.2592 (76),736.2964 (100)	3.695 ± 0.045	0.003 ± 0.001	0.003 ± 0.000	0.003 ± 0.001
6022	6.23	750	Hydroxyferuroyl-di-sinapoylspermidine,putative	ATH02p05039	147.0427 (7), 175.0362 (18),193.0451 (24), 207.0594 (65),250.0997 (34), 264.1103 (11),321.1663 (12), 338.1973 (8),352.2212 (17), 526.2408 (33),544.2575 (91), 545.2682 (9),558.2371 (13), 750.3192 (100)	0.393 ± 0.208	0.003 ± 0.001	0.003 ± 0.000	0.003 ± 0.001

### Metabolic phenotyping of Ds transposon insertion lines

To evaluate the suitability of the MS2T-based method for phytochemical genomics studies, metabolite profiling was conducted using *Ds* insertional mutants of Arabidopsis that were developed for phenome analysis (Kuromori *et al.*, 2004, 2006). First, we analyzed 2-week-old seedlings of all homozygous mutants with transposon insertions in the coding regions of genes encoding UDP-dependent glycosyltransferase (UGT) or methyltransferase. The metabolic profile data for 73 lines (219 samples by triplicate analysis) was acquired within four working days, and a data matrix containing 1808 rows was obtained. The MS2T libraries created above (ATH01p and ATH02p) could tag MS2T data to 604 rows (33%), and 58 rows were annotated using the above-mentioned annotation data. The low coverage of MS2T tagging was due to the lack of root-specific metabolite data in the MS2T libraries.

**Figure 7:**
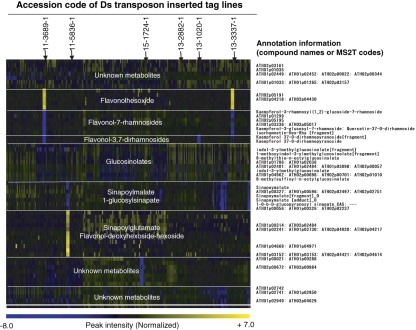
Metabolic profiles of Arabidopsis mutant lines with disruption in putative UDP-dependent glycosyltransferase (UGT) or methyltransferase family genes by insertion of the *Ds* transposon. Two-week-old seedlings of 60 mutant lines and 13 wild-type (F-Nossen) parent lines were analyzed (219 samples in total, *n* =3), and the metabolic profile data were processed to obtain a matrix containing 1808 rows. The log_2_-transformed intensity data are normalized and hierarchically clustered using average linkage methods with the Euclidean distance function. The intensities of 59 peaks in the matrix are represented using a heat map.

A comparison of the metabolic profiles revealed that drastic changes were observed in mutant lines 11-3689-1, 13-3337-1, 13-1020-1 and 11-5836-1 (Figure 7). The functions of the disrupted genes in these lines could easily be ascertained from the changes in metabolites deduced by MS2T-based peak annotation information. For example, the levels of flavonol 7-rhamnoside derivatives were significantly reduced and that of quercetin dihexoside (ATH02p04218, data not shown) was increased in 11-3689-1 and 13-3337-1, suggesting that these mutants lacked the ability to produce 7-*O*-rhamnosyl flavonols. These lines are two mutant alleles of an identical gene, At1g06000, which has recently been identified as encoding UDP-rhamnose:flavonol-7-*O*-rhamnosyltransferase (UGT89C1) (Yonekura-Sakakibara *et al.*, 2007). The metabolite phenotype of 13-1020-1, with a decrease in flavonol-3,7-dirhamnoside, could also be explained by the function of its disrupted gene, UGT78D1 (At1g30530, UDP-rhamnose:flavonol-3-*O*-rhamnosyltransferase) (Jones *et al.*, 2003).

In 11-5836-1 (*Ds* inserted into gene AtUGT84A2, At3g21560), the levels of sinapoylmalate and 1-glucosylsinapate were slightly decreased due to knockout of UGT84A2 that is responsible for the conversion of sinapate to 1-glucosylsinapate (Sinlapadech *et al.*, 2007). In addition, the levels of two metabolites tentatively identified as sinapoylglutamate (ATH01p00314, Figure 6c) and quercetin deoxyhexosyl hexoside (ATH02p04020, data not shown) were increased. This may suggest an inter-connection of those increased metabolites with 1-glucosylsinapate that is affected by disruption of the UGT84A2 gene. A loss-of-metabolite phenotype was found in 15-1724-1 but not the allelic mutant 13-2882-1, indicating that there was no correlation between the phenotype in 15-1724-1 and disruption of the *Ds*-inserted gene (AtUGT85A7, At1g22340) (Woo *et al.*, 2007). No significant metabolic phenotype was observed in other mutants.

## Discussion

One remarkable technical advance achieved by non-targeted metabolic analyses using LC-MS is that a metabolic event occurring in plants can be elucidated by determining a wide range of secondary metabolites, which will assist in formulation of a working hypothesis for further characterization of plant metabolic functions. Although many peaks in metabolite profile data must be annotated for this purpose, they can rarely be annotated using standard compound information (see ‘Compound’ column in Table S3). This situation can be improved if the metabolite peaks are already tagged with MS/MS spectral data prior to the data-mining process. Recently, several MS/MS spectra-based strategies involving flow-injection MS and Fourier transform MS methods have been reported (Beckmann *et al.*, 2008; Cao *et al.*, 2008; Iijima *et al.*, 2008; Overy *et al.*, 2008; Wrona *et al.*, 2005). The methodology was improved in this study by introducing the concept of MS2T and creating MS2T libraries of many known and unknown metabolites that could be used as a basis for peak annotation of LC-MS metabolome data (Figure 2). One of the most significant technical advances of this MS2T-based strategy was that the MS2T libraries were created prior to metabolic profiling analysis; this was achieved by using optimized methods for acquisition of a large amount of MS/MS data. As the MS2T data for most peaks have already been acquired, the MS/MS data acquisition function can be excluded from routine metabolic profiling analysis, which enables high-throughput acquisition of metabolic profiling data (20 min per sample, Figure 7). Once the MS2T libraries have been created, they can be used for annotating data with similar metabolic profiles. Indeed, the MS2T libraries created in this study were used for the annotation of data from *Ds* transposon-tagged lines (Figure 7) as well as the inter-tissue comparison (Figures 3 and 5). Furthermore, the MS2T library can be applied for analysis of metabolic profile data acquired by using other LC-MS methods that employ identical or compatible LC conditions. In addition, it is notable that the entire peak annotation process described in this study was completed in a ‘dry’ lab (Figure 1b), without performing any additional ‘wet’ MS/MS analysis.

### MS2T-based peak annotation

In this study, metabolic profile data were acquired using LC-Q-TOF/MS (Figure 1b, step 1), and the data matrices were generated using MetAlign (Figure 1b, step 2) (de Vos *et al.*, 2007). Each row (peak) in the matrix was annotated using two sets of metabolite-related information, including the standard compound data and the MS2T libraries, by comparing the *m*/*z* and retention time data (Figure 1b, step 3). Consequently, approximately 3% and 50% of the peaks (rows) in the matrix were tentatively annotated and tagged using the standard compound and MS2T data, respectively (Table S3). On the basis of the MS2T data, structural information was assigned by referring to databases of plant metabolites, such as KNApSAcK (Oikawa *et al.*, 2006; Shinbo *et al.*, 2006) and MassBank (Taguchi *et al.*, 2007). However, as the tentative annotation information may contain many false positives, only 2% of the peaks in total were finally annotated despite application of a large amount of data and many databases (Table 1). One of the reasons for this disappointing result is the incomplete MS/MS spectral database of phytochemicals. Interpretation of MS/MS data requires reference spectral data as estimation of the *de novo* structure from the MS/MS spectrum is often difficult even though high-resolution *m*/*z* data are available (Bocker and Rasche, 2008; Werner *et al.*, 2008). Another reason is that there is no existing method to estimate the false-positive ratio in database search results. Because of these technical problems, cross-validation of the annotation data is necessary to obtain plausible annotations; however, many correct annotations are likely to be discarded. This indicates that further development of the informatics basis is required in terms of integration of the MS/MS spectral database of plant secondary metabolites (Baumann *et al.*, 2000; Fredenhagen *et al.*, 2005; Halket *et al.*, 2005; Taguchi *et al.*, 2007; Wishart *et al.*, 2007) and its search algorithm. For this purpose, we are creating a MS/MS spectral database of authentic standards of plant secondary metabolites that are available from MassBank (http://www.massbank.jp/). However, matching of MS/MS spectra poses technical problems because the fragmentation patterns of the MS/MS spectral data depend on the type of mass spectrometer and its operating conditions, especially collision energy (Werner *et al.*, 2008). The cosine product method used in this study, which was originally developed for comparing GC-MS spectra, cannot adequately deal with these problems. To overcome this problem, all the MS/MS spectral data in the MS2T library in this study were obtained using the ‘ramp’ mode, by which fragments detected at various collision energies are combined into one spectrum (Figure 4). In addition, a method termed a ‘spectral motif search’ was developed for searching similar MS/MS spectra from MS2T libraries. Comparison of metabolite structures with these MS/MS spectra allowed us to obtain a ‘spectral motif’, which represents the common structural patterns of neutral losses and fragment ions in a series of metabolites (Figure 4a,b). The spectral motifs are abstract expressions of MS/MS spectra and are partly independent of the nature of the MS/MS spectra, such as the fragment-ion intensities. Although information on neutral loss and fragment ion in MS/MS spectra has been used for metabolite identification, searching MS2T libraries using ‘spectral motifs’ as queries enabled us to identify structurally related metabolites from the metabolic profile data; this technique was then applied for annotation of a series of flavonol glycosides, glucosinolates and hydroxycinnamoylspermidines (Figure 4 and Table 1).

Using these methods, a total of 97 peaks of 48 metabolites in a matrix comprising 1233 rows were identified or tentatively annotated by means of the MS2T method (Table S5). The number of annotatable peaks will increase with further interpretation of the MS2T data, as approximately 600 peaks have already been tagged by MS2Ts. Recently, much effort has been invested in the annotation of metabolites by interpretating MS/MS spectral data. For example, Bottcher *et al.* (2008) reported the annotation of 75 Arabidopsis seed metabolites by manual interpretation of MS/MS spectra. As MS2T libraries of Arabidopsis shoot metabolites have been created, the published information can be used for further annotation of MS2T library data by performing spectral motif searches. It should be noted that most of the annotation information is tentative or involves putative estimation of the metabolite structure; therefore, co-characterization with authentic standards of secondary metabolites prepared from plant extracts is still necessary for rigorous identification of metabolites (Glauser *et al.*, 2008; Ishihara *et al.*, 2006).

### Application of the MS2T-based method for elucidating metabolic events in Arabidopsis

In this study, we demonstrated that the LC-MS profiling technique could elucidate metabolic events in plants to provide a working hypothesis for further characterization of plant metabolic functions by quantitative determination of metabolite levels and MS2T-based peak annotation. The profiling of four distinctive Arabidopsis tissues revealed that the leaves, stems and inflorescence tissues of Arabidopsis have their own unique metabolites (Figure 3); this is probably due to tissue-specific expression of genes responsible for biosynthesis of these metabolites (Schmid *et al.*, 2005). Further, the biosynthesis of two major classes of Arabidopsis secondary metabolites, including flavonoids and glucosinolates, was controlled by the tissue-specific expression of genes responsible for their biosynthesis (Figure 5 and Figure S2). This was also true in the case of tyramine accumulation in inflorescence tissues (Figure 5), which was accompanied by flower tissue-specific expression of a putative tyrosine decarboxylase gene (At4g28680, Figure S2c). Although no role for tyramine or that alkaloid derived from tyramine has been reported in Arabidopsis, the above result suggests that activation of tyramine biosynthesis has a role in the reproductive tissues of Arabidopsis, similar to the reproductive tissue-specific biosynthesis of various tyramine-derived alkaloids in other plant species (Negrel and Martin, 1984; Page, 2005).

In this study, peak annotations by the interpretation of the MS2T data can reveal, at least in part, novel aspects of tissue-specific secondary metabolism in Arabidopsis. For example, a rosette tissue-specific metabolite was putatively concluded to be *p*-coumaroylagmatine (Figure 6a) (von Ropenack *et al.*, 1998). *p*-coumaroylagmatine is a precursor for the biosynthesis of hordatines, which play an important role in resistance to fungal attack in barley seedlings (Ishihara *et al.*, 2002; von Ropenack *et al.*, 1998). Although no hordatine-like metabolites have been detected in healthy Arabidopsis tissues (data not shown), this finding suggests that some biotic stress conditions might stimulate the biosynthesis of similar metabolites in Arabidopsis. A BLASTP search (http://www.tair.org/) revealed that HvACT1, which is responsible for the synthesis of *p*-coumaroylagmatine in barley (Burhenne *et al.*, 2003) (GenBank accession number AB334132) showed the highest homology to AtHCT (At5g48930) of all Arabidopsis genes. AtHCT has already been characterized as an acyltransferase for synthesis of *p*-coumaroylshikimate in the lignin biosynthesis pathway (Hoffmann *et al.*, 2003, 2004), and is highly expressed in the stem tissue as it is required for xylem formation (Figure S2d). This suggests that other acyltransferase genes might be responsible for the rosette leaf-specific biosynthesis of *p*-coumaroylagmatine.

Furthermore, several metabolites specific to inflorescence tissues (Figure 3 and Table 2) were estimated to be hydroxycinnamoylspermidines, such as di-*p*-coumaroylspermidine (Figure 6b). The occurrence of hydroxycinnamoylspermidines in reproductive tissues, e.g. in the pollen of several plant species (Martin-Tanguy *et al.*, 1978; Meurer *et al.*, 1986), and their biological activities (Fixon-Owoo *et al.*, 2003) have been reported; however, their role in the reproductive process has not been investigated genetically or functionally in any plant. Recently, it has been demonstrated that agmatine is the first intermediate of the spermidine biosynthetic pathway from l-arginine in Arabidopsis (Illingworth *et al.*, 2003; Janowitz *et al.*, 2003). These results indicate that tissue-specific synthesis of various types of hydroxycinnamic acid amides from two metabolically related amines in Arabidopsis is probably due to tissue-specific expression of biosynthesis-related acyltransferase genes. These findings facilitate narrowing down of the candidate genes responsible for metabolic functions. For example, evaluation of the expression profiles of 89 genes in the acyltransferase family revealed that several genes showed rosette leaf-specific (e.g. At5g07870, Figure S2e) or pollen-specific (e.g. At4g29440, Figure S2f) expression profiles. This result must be confirmed by the unambiguous identification of metabolites, biochemical characterization of the expressed proteins, and metabolic phenotyping of loss/gain-of-function mutants.

### Link to genetic resources of Arabidosis

Non-targeted metabolic profiling analysis will play an important role in functional genomic studies as it enables metabolic phenotyping of mutants to investigate the functions of disrupted genes *in planta*. Thus, it is believed that high-throughput metabolic phenotyping of a number of mutant lines by non-targeted profiling analysis will reveal novel gene functions without *a priori* knowledge of disrupted genes. The metabolic phenotyping of *Ds* insertion mutants of Arabidopsis demonstrated that the MS2T-based metabolome analysis is an effective tool in terms of high-throughput elucidation of metabolic phenotypes. The clear correlation between the metabolic phenotypes and disrupted genes revealed the gene function *in planta* (Figure 7). As other major changes were not observed in our non-targeted analysis, the functions of these genes were further clarified as specific to those characterized previously.

These results demonstrated that non-targeted metabolic profiling analysis using LC-MS together with the MS2T annotation methods developed in this study could prove to be a useful tool for investigating the novel function of plant secondary metabolites. The developed method is capable of analyzing the metabolic profiles of other plant species, including major crops such as rice and wheat (data not shown), and is also applicable in various fields of metabolomics research. However, a detailed investigation of Arabidopsis to detect functionally and genetically uncharacterized secondary metabolites as a model of other plant species is also important because the various genetic and informatics resources, as well as the ‘omics’ techniques (Hirai *et al.*, 2007; Kuromori *et al.*, 2006; Saito *et al.*, 2008; Tohge *et al.*, 2005; Yonekura-Sakakibara *et al.*, 2007), enable us to perform phytochemical genomics studies to reveal novel functions of plant secondary metabolism.

### Availability of source programs

The data and programs produced in this study are freely available on the Platform for Riken Metabolomics (PRIMe) website (http://prime.psc.riken.jp/lcms/).

## Experimental procedures

### Chemicals

All the chemicals used in this study were purchased from Tokyo Kasei (http://www.tciamerica.com), Sigma-Aldrich (http://www.sigmaaldrich.com/), Wako Pure Chemical (http://wako-chem.co.jp/english/), Nacalai Tesque (http://www.nacalai.co.jp/en/index) and AnalytiCon Discovery GmbH (http://www.ac/discovery.com/english/go.html). Indole-3-ylmethylglucosinolate, 1-methoxyindole-3-ylmethylglucosinolate and 4-methoxyindole-3-ylmethylglucosinolate were prepared as previously described (Ishihara *et al.*, 2006). A total of 29 metabolites derived from Arabidopsis were isolated from whole plants of *A. thaliana* (Nakabayashi *et al.*, unpublished results).

## Plant materials

Seedlings of *Arabidopsis thaliana* (Col-0) were grown in pots containing soil at 20°C with a 16 h daily photoperiod. Six weeks after germination, the 12th or 13th expanded rosette leaves (rosette leaf), the 1st and 2nd expanded cauline leaves (cauline leaf), the upper part of the inflorescence (inflorescence), and first internode tissues (stem) were collected from eight individual Arabidopsis plants at stage 6.3 (Boyes *et al.*, 2001) and stored at −80°C until use. For metabolic phenotyping of *Ds* transposon insertion lines (Kuromori *et al.*, 2004, 2006), 60 lines of homozygous seeds were grown on the half-strength MS medium plates at 20°C with a 16 h daily photoperiod. Two weeks after germination, whole tissues of 20 seedlings were collected, weighed, and stored at −80°C.

### Non-targeted metabolic profiling analysis using LC-ESI-MS

The frozen tissues were homogenized in five volumes of 80% aqueous methanol containing 0.5 mg l^−1^ lidocaine and d-camphor sulfonic acid (Tokyo Kasei) using a mixer mill (MM 300, Retsch, http://www.retsch.com) with a zirconia bead for 6 min at 20 Hz. Following centrifugation of 15 000 ***g*** for 10 min and filtration (Ultrafree-MC, 0.2 μm pore size; Millipore, http://www.millipore.com the sample extracts (2 μl) were analyzed using an LC-MS system equipped with an electrospray ionization (ESI) interface (HPLC, Waters Acquity UPLC system; MS, Waters Q-Tof Premier, http://www.waters.com). The analytical conditions were as follows. HPLC: column, Acquity bridged ethyl hybrid (BEH) C18 (pore size 1.7 μm, length 2.1 × 100 mm, Waters); solvent system, acetonitrile (0.1% formic acid):water (0.1% formic acid); gradient program, 1 : 99 v/v at 0 min, 1 : 99 v/v at 0.1 min, 99.5 : 0.5 at 15.5 min, 99.5 : 0.5 at 17.0 min, 1 : 99 v/v at 17.1 min and 1 : 99 at 20 min; flow rate, 0.3 ml min^−1^; temperature, 38°C; MS detection: capillary voltage, +3.0 keV; cone voltage, 22.5 V; source temperature, 120°C; desolvation temperature, 450°C; cone gas flow, 50 l h^−1^; desolvation gas flow, 800 l h^−1^; collision energy, 2 V; detection mode, scan (*m*/*z* 100–2000; dwell time 0.45 sec; interscan delay 0.05 sec, centroid). The scans were repeated for 19.5 min in a single run. The data were recorded using MassLynx version 4.1 software (Waters).

### Data processing and MS2T-based peak annotation

The data matrix was generated from the metabolic profile data using MetAlign software (de Vos *et al.*, 2007) and processed using in-house software written in Perl/Tk (‘N toolbox’, Appendix S3). Detailed methods for processing and interpretation of the MS2T data are described in Appendix S2. The processed data matrix was analyzed using MeV4.0 (TIGR, http://www.tm4.org) Saeed *et al.*, 2003, 2006).

### MS2T data acquisition

The sample extracts prepared by the method above (2 μl) were subjected to the same LC-Q-TOF-MS system operated under the same conditions mentioned above, except for the following changes: gradient program, 1 : 99 v/v at 0 min, 1 : 99 v/v at 0.2 min, 99.5 : 0.5 at 31 min, 99.5 : 0.5 at 34.0 min, 1 : 99 v/v at 34.2 min and 1 : 99 at 40 min; flow rate 0.15 ml min^−1^; survey detection mode for MS detection. In this mode, following acquisition of the MS spectrum (*m*/*z* 100–1000; dwell time 0.45 sec, inter-scan delay 0.05 sec), the MS/MS data of the most abundant ions were automatically obtained (*m*/*z* 50–1000; dwell time 2.5 sec; inter-scan delay 0.5 sec, data acquisition, centroid mode; collision energy ramped from 5 to 60 V). The mass/charge ratio (*m*/*z*) was calibrated using the lock-mass function with leucine enkephalin. The analyses were repeated 25 times by shifting the *m*/*z* ranges of the target ion selection window for the MS/MS analysis (*m*/*z* 100–160, 130–190, 160–220 … 880–940, 940–1000). The data were converted into ASCII format using DataBridge (Waters). The information in each MS/MS spectrum was formatted to the MS2T libraries using in-house Perl scripts. Low-intensity signals of fewer than 5 counts/sec were discarded in this process. The original retention time values were divided by two to compensate for the difference in peak elution conditions.

## Supporting information

**Figure S1.** Representative LC-Q-TOF/MS chromatogram of a sample extract derived from a rosette leaf of a 6-week-old Arabidopsis seedling recorded in positive ion mode.

**Figure S2.** Tissue-dependent expression profiles of genes responsible for the biosynthesis of Arabidopsis secondary metabolites.

**Figure S3.** MS/MS spectra of MS2Ts and deduced fragmentation schemes of ATH01p05697 (p-coumaroylagmatine, putative), ATH02p02987 (di-p-coumarolyspermidine, putative) and TH01p00314 (sinapoylglutamate, putative).

**Table S1.** MS2T libraries (ATH01p and ATH02p) obtained from the shoot and inflorescence tissues of A. thaliana.

**Table S2.** Original matrix of metabolite profile data produced by METALIGN.

**Table S3.** Processed and annotated data matrix.

**Table S4.** Retention times and m/z data for 280 standard compounds (standard compound list).

**Table S5.** Curated annotation list.

**Appendix S1.** Quality evaluation of MS2T data library.

**Appendix S2.** Detailed procedure for processing of metabolic profile data.

**Appendix S3.** Source codes (Perl/Tk) of the N toolbox (‘Nprefilter. pl’, ‘Nnormalizer.pl’, ‘Nfilter.pl’, ‘Nisotoperemover.pl’, ‘Nannotator. pl’ and ‘Nmotifsearch.pl’).

## References

[b1] Baumann C, Cintora MA, Eichler M, Lifante E, Cooke M, Przyborowska A, Halket JM (2000). A library of atmospheric pressure ionization daughter ion mass spectra based on wideband excitation in an ion trap mass spectrometer. Rapid Commun. Mass Spectrom..

[b2] Beckmann M, Parker D, Enot DP, Duval E, Draper J (2008). High-throughput, nontargeted metabolite fingerprinting using nominal mass flow injection electrospray mass spectrometry. Nat. Protoc..

[b3] Bino RJ, Hall RD, Fiehn O (2004). Potential of metabolomics as a functional genomics tool. Trends Plant Sci..

[b4] Bocker S, Rasche F (2008). Towards de novo identification of metabolites by analyzing tandem mass spectra. Bioinformatics.

[b5] Bottcher C, von Roepenack-Lahaye E, Schmidt J, Schmotz C, Neumann S, Scheel D, Clemens S (2008). Metabolome analysis of biosynthetic mutants reveals diversity of metabolic changes and allows identification of a large number of new compounds in *Arabidopsis thaliana*. Plant Physiol..

[b6] Boyes DC, Zayed AM, Ascenzi R, McCaskill AJ, Hoffman NE, Davis KR, Gorlach J (2001). Growth stage-based phenotypic analysis of Arabidopsis: a model for high throughput functional genomics in plants. Plant Cell.

[b7] Broeckling CD, Huhman DV, Farag MA, Smith JT, May GD, Mendes P, Dixon RA, Sumner LW (2005). Metabolic profiling of *Medicago truncatula* cell cultures reveals the effects of biotic and abiotic elicitors on metabolism. J. Exp. Bot..

[b8] Brown PD, Tokuhisa JG, Reichelt M, Gershenzon J (2003). Variation of glucosinolate accumulation among different organs and developmental stages of *Arabidopsis thaliana*. Phytochemistry.

[b9] Burhenne K, Kristensen BK, Rasmussen SK (2003). A new class of *N*-hydroxycinnamoyltransferases. Purification, cloning and expression of a barley agmatine coumaroyltransferase (EC 2.3.1.64). J. Biol. Chem..

[b10] Cao M, Koulman A, Johnson LJ, Lane GA, Rasmussen S (2008). Advanced data-mining strategies for the analysis of direct-infusion ion trap mass spectrometry data from the association of perennial ryegrass with its endophytic fungus, *Neotyphodium lolii*. Plant Physiol..

[b11] Dettmer K, Aronov PA, Hammock BD (2007). Mass spectrometry-based metabolomics. Mass Spectrom. Rev..

[b12] Dunn WB (2008). Current trends and future requirements for the mass spectrometric investigation of microbial, mammalian and plant metabolomes. Phys. Biol..

[b13] Farag MA, Huhman DV, Lei Z, Sumner LW (2007). Metabolic profiling and systematic identification of flavonoids and isoflavonoids in roots and cell suspension cultures of *Medicago truncatula* using HPLC-UV-ESI-MS and GC-MS. Phytochemistry.

[b14] Farag MA, Huhman DV, Dixon RA, Sumner LW (2008). Metabolomics reveals novel pathways and differential mechanistic and elicitor-specific responses in phenylpropanoid and isoflavonoid biosynthesis in *Medicago truncatula* cell cultures. Plant Physiol..

[b15] Fixon-Owoo S, Levasseur F, Williams K, Sabado TN, Lowe M, Klose M, Joffre Mercier A, Fields P, Atkinson J (2003). Preparation and biological assessment of hydroxycinnamic acid amides of polyamines. Phytochemistry.

[b16] Fredenhagen A, Derrien C, Gassmann E (2005). An MS/MS library on an ion-trap instrument for efficient dereplication of natural products. Different fragmentation patterns for [M + H]+ and [M + Na]+ ions. J. Nat. Prod..

[b17] Glauser G, Guillarme D, Grata E, Boccard J, Thiocone A, Carrupt PA, Veuthey JL, Rudaz S, Wolfender JL (2008). Optimized liquid chromatography-mass spectrometry approach for the isolation of minor stress biomarkers in plant extracts and their identification by capillary nuclear magnetic resonance. J. Chromatogr. A.

[b18] Grata E, Boccard J, Guillarme D, Glauser G, Carrupt PA, Farmer EE, Wolfender JL, Rudaz S (2008). UPLC-TOF-MS for plant metabolomics: a sequential approach for wound marker analysis in *Arabidopsis thaliana*. J. Chromatogr. B.

[b19] Halket JM, Waterman D, Przyborowska AM, Patel RK, Fraser PD, Bramley PM (2005). Chemical derivatization and mass spectral libraries in metabolic profiling by GC/MS and LC/MS/MS. J. Exp. Bot..

[b20] Hansen BG, Kliebenstein DJ, Halkier BA (2007). Identification of a flavin-monooxygenase as the *S*-oxygenating enzyme in aliphatic glucosinolate biosynthesis in Arabidopsis. Plant J..

[b21] Hernandez P, Muller M, Appel RD (2006). Automated protein identification by tandem mass spectrometry: issues and strategies. Mass Spectrom. Rev..

[b22] Hirai MY, Sugiyama K, Sawada Y (2007). Omics-based identification of Arabidopsis Myb transcription factors regulating aliphatic glucosinolate biosynthesis. Proc. Natl Acad. Sci. USA.

[b23] Hoffmann L, Maury S, Martz F, Geoffroy P, Legrand M (2003). Purification, cloning and properties of an acyltransferase controlling shikimate and quinate ester intermediate in phenylpropanoid metabolism. J. Biol. Chem..

[b24] Hoffmann L, Besseau S, Geoffroy P, Ritzenthaler C, Meyer D, Lapierre C, Pollet B, Legrand M (2004). Silencing of hydroxycinnamoyl-coenzyme A shikimate/quinate hydroxycinnamoyltransferase affects phenylpropanoid biosynthesis. Plant Cell.

[b25] Iijima Y, Nakamura Y, Ogata Y (2008). Metabolite annotations based on the integration of mass spectral information. Plant J..

[b26] Illingworth C, Mayer MJ, Elliott K, Hanfrey C, Walton NJ, Michael AJ (2003). The diverse bacterial origins of the Arabidopsis polyamine biosynthetic pathway. FEBS Lett..

[b27] Ishihama Y (2005). Proteomic LC-MS systems using nanoscale liquid chromatography with tandem mass spectrometry. J. Chromatogr. A.

[b28] Ishihara A, Ogura Y, Tebayashi S, Iwamura H (2002). Jasmonate-induced changes in flavonoid metabolism in barley (*Hordeum vulgare*) leaves. Biosci. Biotechnol. Biochem..

[b29] Ishihara A, Asada Y, Takahashi Y, Yabe N, Komeda Y, Nishioka T, Miyagawa H, Wakasa K (2006). Metabolic changes in *Arabidopsis thaliana* expressing the feedback-resistant anthranilate synthase α subunit gene *OASA1D*. Phytochemistry.

[b30] Janowitz T, Kneifel H, Piotrowski M (2003). Identification and characterization of plant agmatine iminohydrolase, the last missing link in polyamine biosynthesis of plants. FEBS Lett..

[b31] Jones P, Messner B, Nakajima J, Schaffner AR, Saito K (2003). UGT73C6 and UGT78D1, glycosyltransferases involved in flavonol glycoside biosynthesis in *Arabidopsis thaliana*. J. Biol. Chem..

[b32] Jonsson P, Johansson ES, Wuolikainen A, Lindberg J, Schuppe-Koistinen I, Kusano M, Sjostrom M, Trygg J, Moritz T, Antti H (2006). Predictive metabolite profiling applying hierarchical multivariate curve resolution to GC-MS data-a potential tool for multi-parametric diagnosis. J. Proteome Res..

[b33] Katajamaa M, Oresic M (2005). Processing methods for differential analysis of LC/MS profile data. BMC Bioinformatics.

[b34] Keurentjes JJ, Fu J, de Vos CH, Lommen A, Hall RD, Bino RJ, van der Plas LH, Jansen RC, Vreugdenhil D, Koornneef M (2006). The genetics of plant metabolism. Nat. Genet..

[b35] Kim JK, Bamba T, Harada K, Fukusaki E, Kobayashi A (2007). Time-course metabolic profiling in *Arabidopsis thaliana* cell cultures after salt stress treatment. J. Exp. Bot..

[b36] Kopka J (2006). Current challenges and developments in GC-MS based metabolite profiling technology. J. Biotechnol..

[b37] Kuromori T, Hirayama T, Kiyosue Y, Takabe H, Mizukado S, Sakurai T, Akiyama K, Kamiya A, Ito T, Shinozaki K (2004). A collection of 11 800 single-copy *Ds* transposon insertion lines in *Arabidopsis*. Plant J..

[b38] Kuromori T, Wada T, Kamiya A (2006). A trial of phenome analysis using 4000 *Ds*-insertional mutants in gene-coding regions of Arabidopsis. Plant J..

[b39] Lisec J, Schauer N, Kopka J, Willmitzer L, Fernie AR (2006). Gas chromatography mass spectrometry-based metabolite profiling in plants. Nat. Protoc..

[b40] Martin-Tanguy J, Cabanne F, Perdrizet E, Martin C (1978). The distribution of hydroxycinnamic acid amides in flowering plants. Phytochemistry.

[b41] Meissner D, Albert A, Bottcher C, Strack D, Milkowski C (2008). The role of UDP-glucose:hydroxycinnamate glucosyltransferases in phenylpropanoid metabolism and the response to UV-B radiation in *Arabidopsis thaliana*. Planta.

[b42] Messerli G, Partovi Nia V, Trevisan M, Kolbe A, Schauer N, Geigenberger P, Chen J, Davison AC, Fernie AR, Zeeman SC (2007). Rapid classification of phenotypic mutants of Arabidopsis via metabolite fingerprinting. Plant Physiol..

[b43] Meurer B, Wray V, Grotjahn L, Wiermann R, Strack D (1986). Hydroxycinnamic acid spermidine amides from pollen of *Corylus avellena* L. Phytochemistry.

[b44] Mintz-Oron S, Mandel T, Rogachev I (2008). Gene expression and metabolism in tomato fruit surface tissues. Plant Physiol..

[b45] Moco S, Bino RJ, Vorst O, Verhoeven HA, de Groot J, van Beek TA, Vervoort J, de Vos CH (2006). A liquid chromatography-mass spectrometry-based metabolome database for tomato. Plant Physiol..

[b46] Moco S, Bino RJ, de Vos CH, Vervoort J (2007a). Metabolomics technologies and metabolite identification. Trends Anal. Chem..

[b47] Moco S, Capanoglu E, Tikunov Y, Bino RJ, Boyacioglu D, Hall RD, Vervoort J, de Vos CH (2007b). Tissue specialization at the metabolite level is perceived during the development of tomato fruit. J. Exp. Bot..

[b48] Negrel J, Martin C (1984). The biosynthesis of feruloyltyramine in *Nicotiana tabacum*. Phytochemistry.

[b49] Oikawa A, Nakamura Y, Ogura T, Kimura A, Suzuki H, Sakurai N, Shinbo Y, Shibata D, Kanaya S, Ohta D (2006). Clarification of pathway-specific inhibition by Fourier transform ion cyclotron resonance/mass spectrometry-based metabolic phenotyping studies. Plant Physiol..

[b50] Overy DP, Enot DP, Tailliart K, Jenkins H, Parker D, Beckmann M, Draper J (2008). Explanatory signal interpretation and metabolite identification strategies for nominal mass FIE-MS metabolite fingerprints. Nat. Protoc..

[b51] Page JE (2005). Silencing nature's narcotics: metabolic engineering of the opium poppy. Trends Biotechnol..

[b52] Rochfort SJ, Trenerry VC, Imsic M, Panozzo J, Jones R (2008). Class targeted metabolomics: ESI ion trap screening methods for glucosinolates based on MSn fragmentation. Phytochemistry.

[b53] von Roepenack-Lahaye E, Degenkolb T, Zerjeski M, Franz M, Roth U, Wessjohann L, Schmidt J, Scheel D, Clemens S (2004). Profiling of Arabidopsis secondary metabolites by capillary liquid chromatography coupled to electrospray ionization quadrupole time-of-flight mass spectrometry. Plant Physiol..

[b54] von Ropenack E, Parr A, Schulze-Lefert P (1998). Structural analyses and dynamics of soluble and cell wall-bound phenolics in a broad spectrum resistance to the powdery mildew fungus in barley. J. Biol. Chem..

[b55] Saeed AI, Sharov V, White J (2003). TM4: a free, open-source system for microarray data management and analysis. BioTechniques.

[b56] Saeed AI, Bhagabati NK, Braisted JC, Liang W, Sharov V, Howe EA, Li J, Thiagarajan M, White JA, Quackenbush J (2006). TM4 microarray software suite. Methods Enzymol..

[b57] Saito K, Hirai MY, Yonekura-Sakakibara K (2008). Decoding genes with coexpression networks and metabolomics –‘majority report by precogs’. Trends Plant Sci..

[b58] Schauer N, Semel Y, Roessner U (2006). Comprehensive metabolic profiling and phenotyping of interspecific introgression lines for tomato improvement. Nat. Biotechnol..

[b59] Schliemann W, Ammer C, Strack D (2007). Metabolite profiling of mycorrhizal roots of *Medicago truncatula*. Phytochemistry.

[b60] Schmid M, Davison TS, Henz SR, Pape UJ, Demar M, Vingron M, Scholkopf B, Weigel D, Lohmann JU (2005). A gene expression map of *Arabidopsis thaliana* development. Nat. Genet..

[b61] Shinbo Y, Nakamura Y, Altaf-Ul-Amin M, Asahi H, Kurokawa K, Arita M, Saito K, Ohta D, Shibata D, Kanaya S, Saito K, Dixon RA, Willmitzer L (2006). KNApSAcK: a comprehensive species–metabolite relationship database. Biotechnology in Agriculture and Forestry 57. Plant Metabolomics.

[b62] Sinlapadech T, Stout J, Ruegger MO, Deak M, Chapple C (2007). The hyper-fluorescent trichome phenotype of the *brt1* mutant of Arabidopsis is the result of a defect in a sinapic acid:UDPG glucosyltransferase. Plant J..

[b63] Smith CA, Want EJ, O’Maille G, Abagyan R, Siuzdak G (2006). XCMS: processing mass spectrometry data for metabolite profiling using nonlinear peak alignment, matching, and identification. Anal. Chem..

[b64] Soga T, Baran R, Suematsu M (2006). Differential metaolomics reveals ophthalmic acid as an oxidative stress biomarker indicating hepatic glutathione consumption. J. Biol. Chem..

[b65] Suzuki H, Sasaki R, Ogata Y (2007). Metabolic profiling of flavonoids in *Lotus japonicus* using liquid chromatography Fourier transform ion cyclotron resonance mass spectrometry. Phytochemistry.

[b66] Taguchi R, Nishijima M, Shimizu T (2007). Basic analytical systems for lipidomics by mass spectrometry in Japan. Methods Enzymol..

[b67] Takahashi H, Kai K, Shinbo Y, Tanaka K, Ohta D, Oshima T, Altaf-Ul-Amin M, Kurokawa K, Ogasawara N, Kanaya S (2008). Metabolomics approach for determining growth-specific metabolites based on Fourier transform ion cyclotron resonance mass spectrometry. Anal. Bioanal. Chem..

[b68] Tikunov Y, Lommen A, de Vos CH, Verhoeven HA, Bino RJ, Hall RD, Bovy AG (2005). A novel approach for nontargeted data analysis for metabolomics. Large-scale profiling of tomato fruit volatiles. Plant Physiol..

[b69] Tohge T, Nishiyama Y, Hirai MY (2005). Functional genomics by integrated analysis of metabolome and transcriptome of Arabidopsis plants over-expressing an MYB transcription factor. Plant J..

[b70] Tohge T, Yonekura-Sakakibara K, Niida R, Watanabe-Takahashi A, Saito K (2007). Phytochemical genomics in *Arabidopsis thaliana*: a case study for functional identification of flavonoid biosynthesis genes. Pure Appl. Chem..

[b71] Villas-Boas SG, Mas S, Akesson M, Smedsgaard J, Nielsen J (2005). Mass spectrometry in metabolome analysis. Mass Spectrom. Rev..

[b72] de Vos CH, Moco S, Lommen A, Keurentjes JJ, Bino RJ, Hall RD (2007). Untargeted large-scale plant metabolomics using liquid chromatography coupled to mass spectrometry. Nat. Protoc..

[b73] Wagner C, Sefkow M, Kopka J (2003). Construction and application of a mass spectral and retention time index database generated from plant GC/EI-TOF-MS metabolite profiles. Phytochemistry.

[b74] Werner E, Heilier JF, Ducruix C, Ezan E, Junot C, Tabet JC (2008). Mass spectrometry for the identification of the discriminating signals from metabolomics: current status and future trends. J. Chromatogr. B.

[b75] Wiklund S, Johansson E, Sjostrom L, Mellerowicz EJ, Edlund U, Shockcor JP, Gottfries J, Moritz T, Trygg J (2008). Visualization of GC/TOF-MS-based metabolomics data for identification of biochemically interesting compounds using OPLS class models. Anal. Chem..

[b76] Wishart DS, Tzur D, Knox C (2007). HMDB: the human metabolome database. Nucleic Acids Res..

[b77] Woo HH, Jeong BR, Hirsch AM, Hawes MC (2007). Characterization of Arabidopsis AtUGT85A and AtGUS gene families and their expression in rapidly dividing tissues. Genomics.

[b78] Wrona M, Mauriala T, Bateman KP, Mortishire-Smith RJ, O’Connor D (2005). ‘All-in-one’ analysis for metabolite identification using liquid chromatography/hybrid quadrupole time-of-flight mass spectrometry with collision energy switching. Rapid Commun. Mass Spectrom..

[b79] Yonekura-Sakakibara K, Tohge T, Niida R, Saito K (2007). Identification of a flavonol 7-*O*-rhamnosyltransferase gene determining flavonoid pattern in Arabidopsis by transcriptome coexpression analysis and reverse genetics. J. Biol. Chem..

[b80] Youhnovski N, Werner C, Hesse M (2001). *N*,*N′*,*N″*-triferuloylspermidine, a new UV absorbing polyamine derivative from pollen of *Hippeastrum* x*hortorum*. Z. Naturforsch..

