# Supplementary material for: MS/MS spectral tag-based annotation of non-targeted profile of plant secondary metabolites
Source: Plant J. 2008 Nov 11;57(3):555–77. doi: 10.1111/j.1365-313X.2008.03705.x (PMC2667644; doi:10.1111/j.1365-313X.2008.03705.x)
Supplement: Supplementary file 1 [file tpj0057-0555-SD1.pdf]

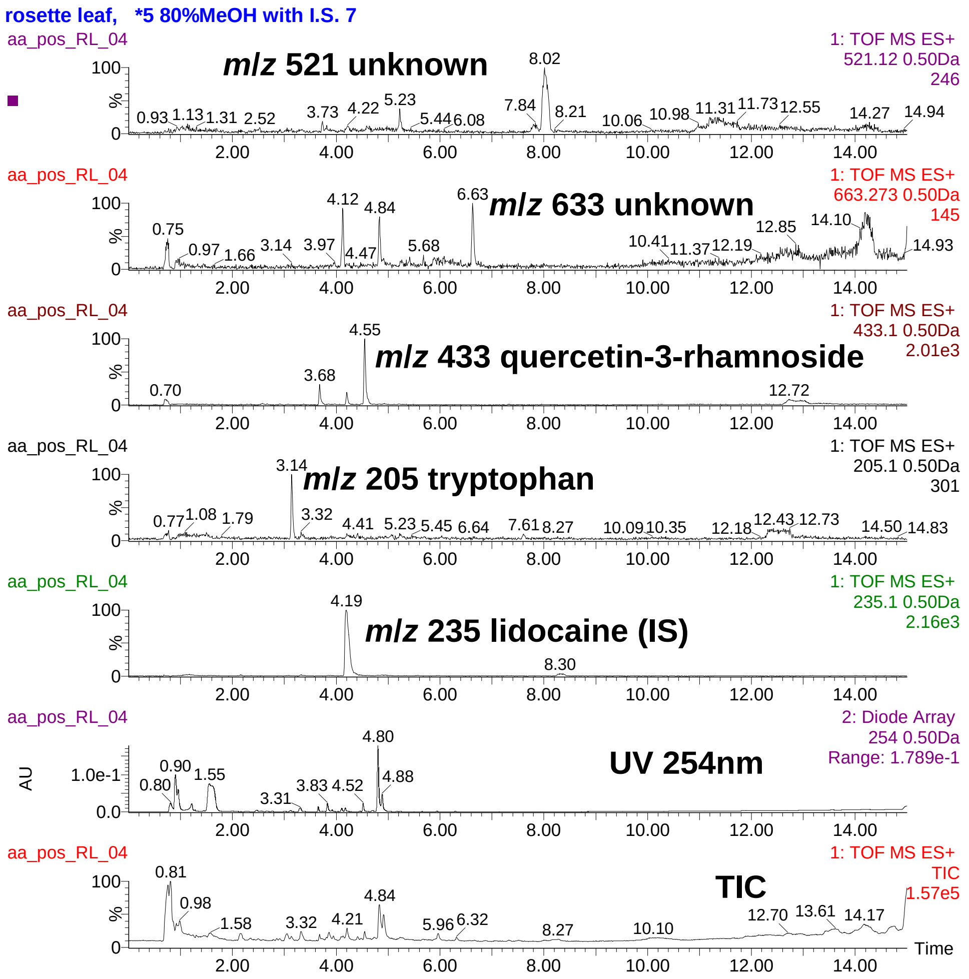

Supplemental Figure S1 Representative LC-QTOFMS chromatogram of the sample extract derived from the rosette leaf of six-week-old Arabidopsis seedling recorded in the positive ion mode. The total ion chromatogram (TIC), UV chromatogram recorded at 254 nm (UV 254 nm) and selected ion chromatograms of identified and unknown metabolites are shown.
