# Supplementary material for: MS/MS spectral tag-based annotation of non-targeted profile of plant secondary metabolites
Source: Plant J. 2008 Nov 11;57(3):555–77. doi: 10.1111/j.1365-313X.2008.03705.x (PMC2667644; doi:10.1111/j.1365-313X.2008.03705.x)

**Supplemental Figure S2** The tissue-dependent expression profiles of genes responsible for the biosynthesis of Arabidopsis secondary metabolites. The expression data were visualized by using the Arabidopsis e-FP Browser (http://bbc.botany.utoronto.ca/efp/cgi-bin/efpWeb.cgi) produced by the Bio-Array Resource for Arabidopsis Functional Genomics (BAR).

a) Expression profile of S-oxygenating enzyme gene (At1g65860)

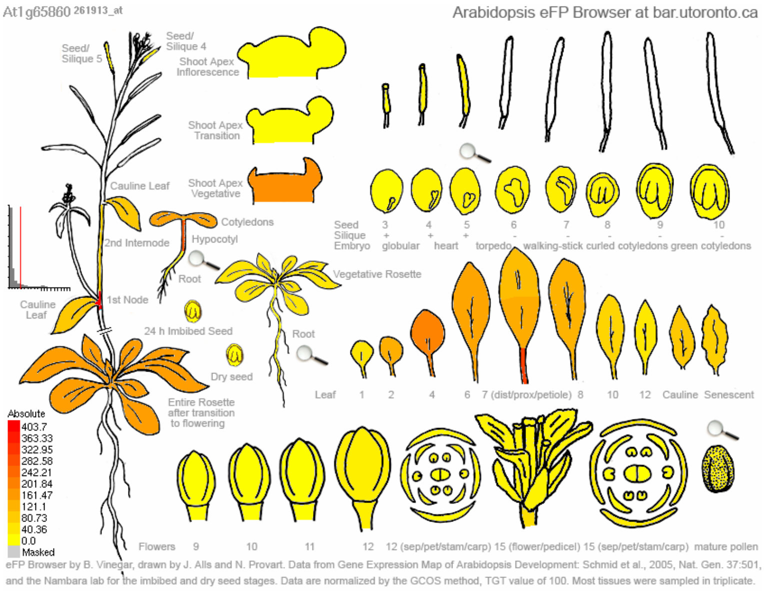

b) Expression profile of *omt1* gene (At5g54160)

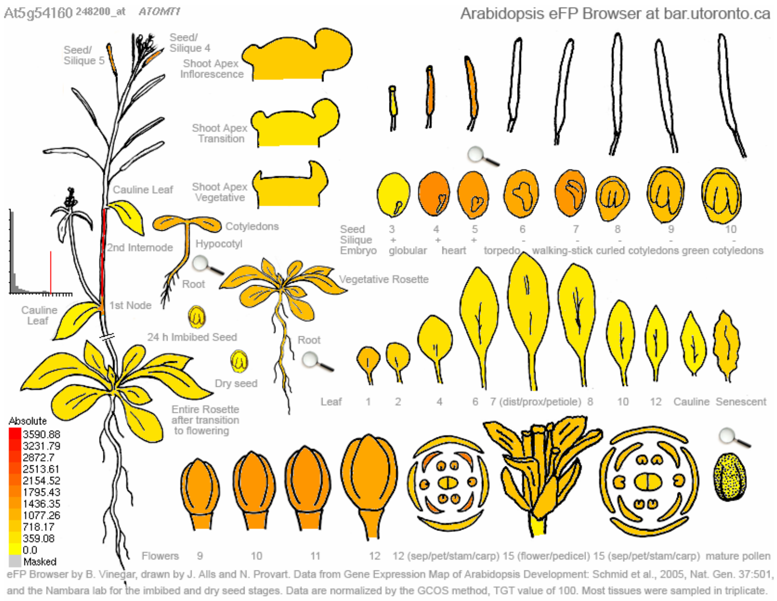


c) Expression profile of putative tyrosine decarboxylase gene (At4g28680)

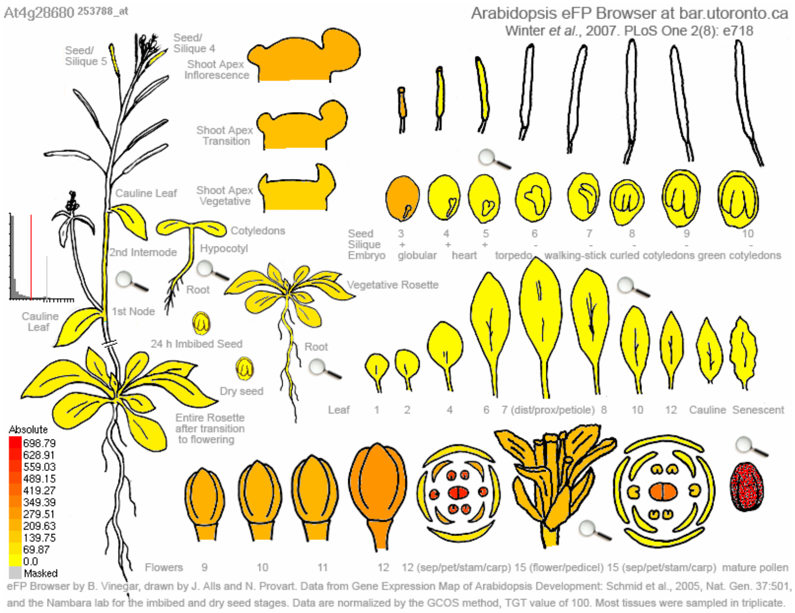

d) Expression profile of a hydroxycinnamoyl transferase (AtHCT) gene (At5g48930)

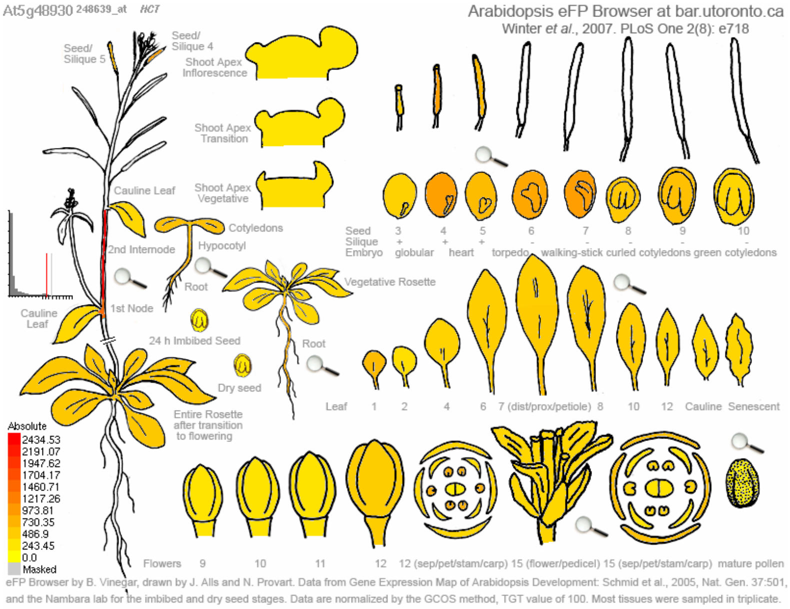


e) Expression profile of a putative hydroxycinnamoyl transferase gene highly expressed in rosette leaf (At5g07870)

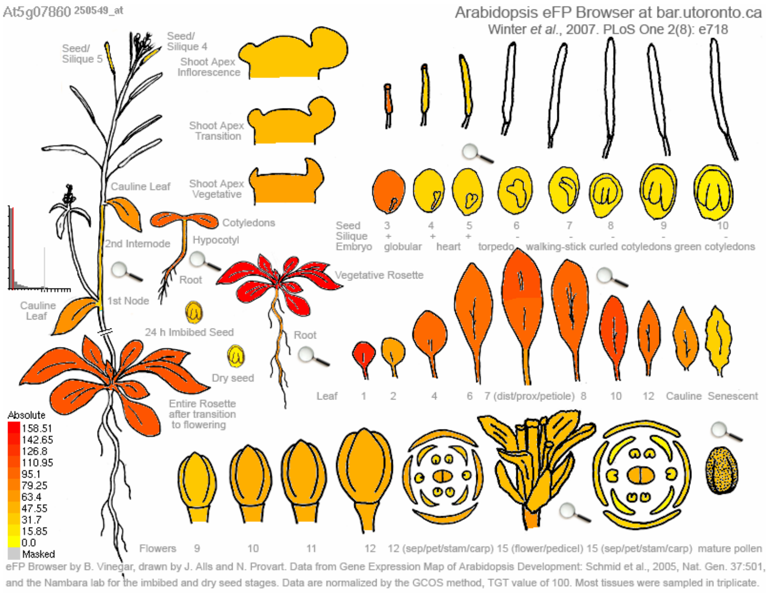

f) Expression profile of a putative hydroxycinnamoyl transferase gene specifically expressed in mature pollen (At4g29440)

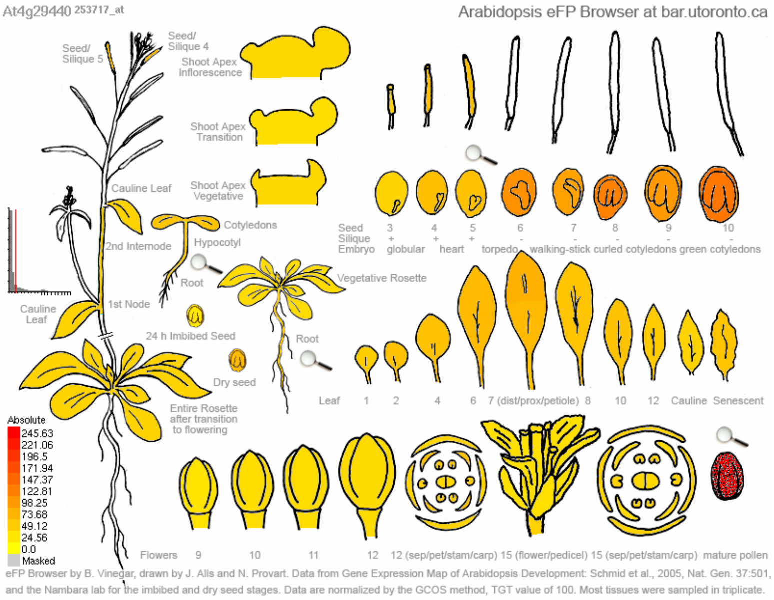

Supplement: Supplementary file 2 [file tpj0057-0555-SD2.pdf]
