# Supplementary material for: MS/MS spectral tag-based annotation of non-targeted profile of plant secondary metabolites
Source: Plant J. 2008 Nov 11;57(3):555–77. doi: 10.1111/j.1365-313X.2008.03705.x (PMC2667644; doi:10.1111/j.1365-313X.2008.03705.x)

Supplemental figure S3 MS/MS spectra of MS2Ts and deduced fragmentation schemes of (a) ATH01p05697, p-coumaroylagmatine, putative, (b) ATH02p02987, di-*p*-coumarolyspermidine, putative, and (c) ATH01p00314, sinapoylglutamate, putative.

(a) ATH01p05697, *p*-coumaroylagmatine

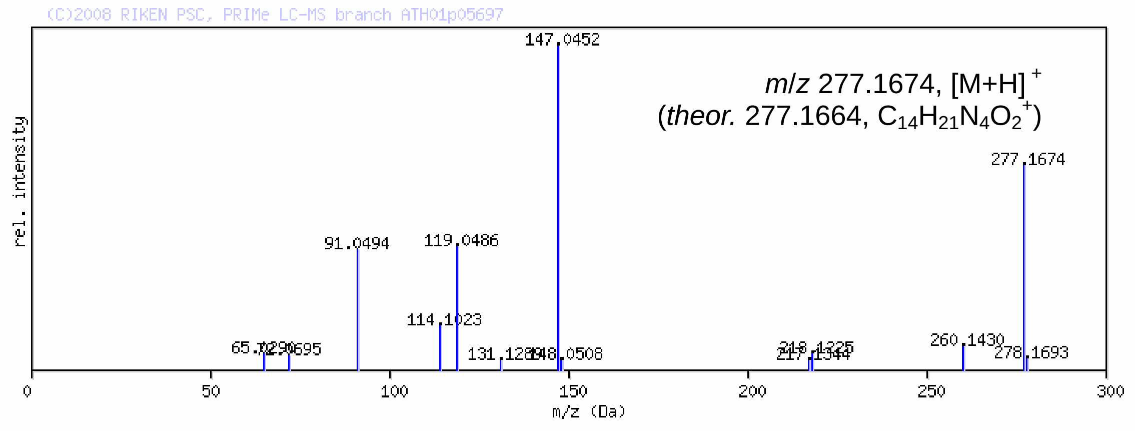

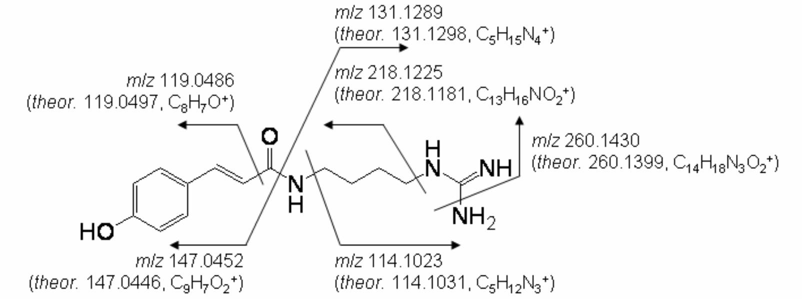

(b) ATH02p02987, di-*p*-coumaroylspermidine

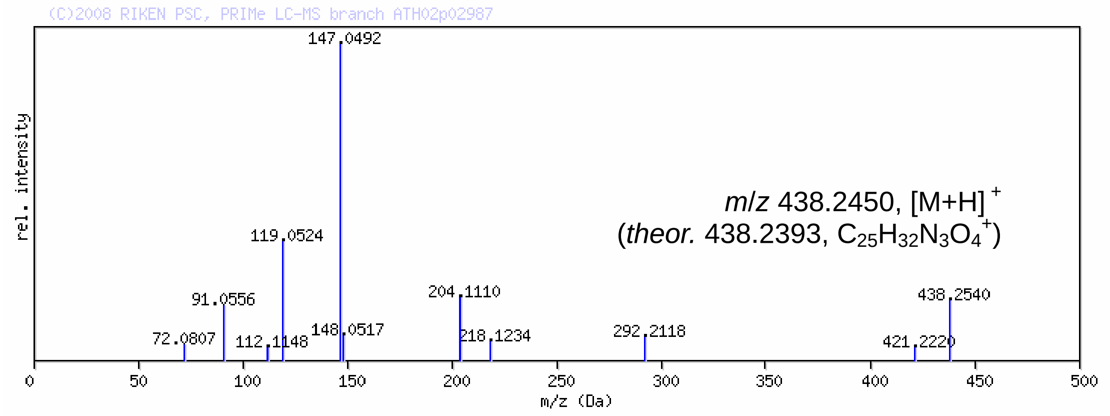

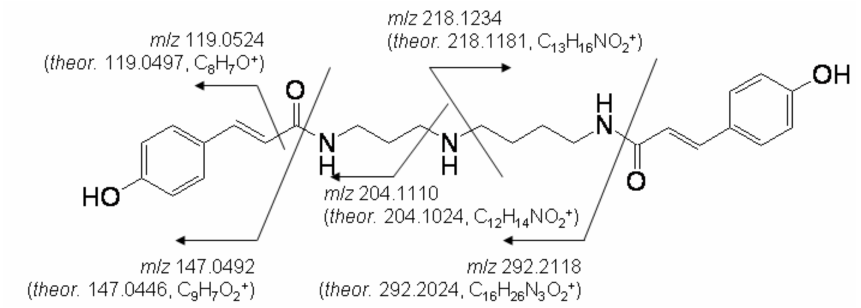

(c) ATH01p00314, sinapoylglutamate

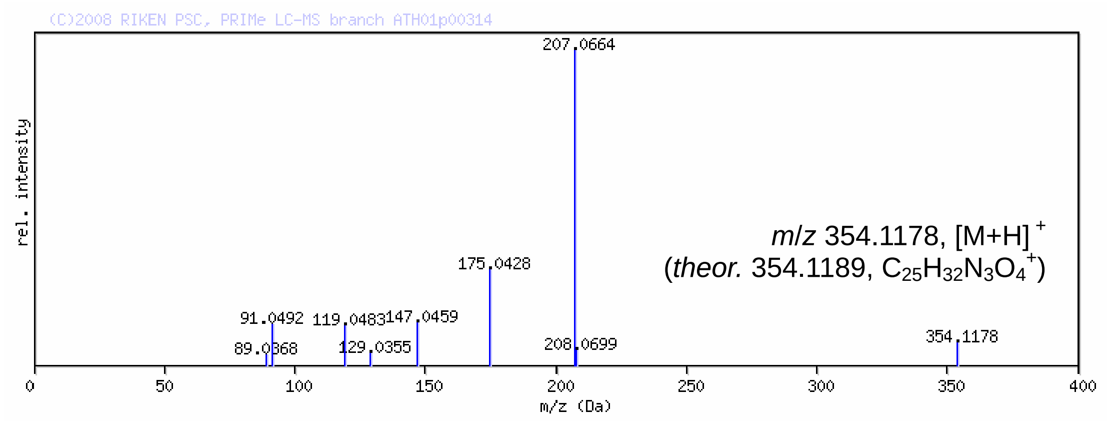

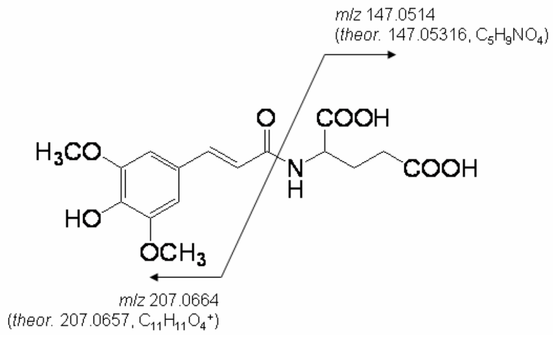

Supplement: Supplementary file 3 [file tpj0057-0555-SD3.pdf]
