# Supplementary material for: MS/MS spectral tag-based annotation of non-targeted profile of plant secondary metabolites
Source: Plant J. 2008 Nov 11;57(3):555–77. doi: 10.1111/j.1365-313X.2008.03705.x (PMC2667644; doi:10.1111/j.1365-313X.2008.03705.x)
Supplement: Supplementary file 15 [file tpj0057-0555-SD15.doc]

**Supplemental text S1** Quality evaluation of MS2T data library.

*Accuracy of m/z values*

High-resolution m/z data of fragment ion signals are one of the most valuable information for the interpretation of MS/MS spectral data recorded in the MS2T library. Although the mass spectrometer was continuously calibrated using the LockSpray apparatus during the experiment, the m/z values inevitably contain some errors. In order to estimate the accuracy of the m/z values, 14 MS2T accessions derived from the protonated molecules of kaempferol-3-glucoside-7-rhamnoside ([M + H]^+^, m/z 595.1) were selected from the two MS2T libraries created in the study (ATH01p and ATH02p). In these MS/MS spectral data, two representative fragment ions derived from keampferol aglycone ([C_15_H_11_O_6_]^+^, m/z 287.0556 (theoretical)) and keampferol-7-rhamnoside ([C_21_H_21_O_10_]^+^, m/z 433.1135 (theoretical)) were observed. A comparison between these theoretical and observed values revealed that the standard deviations (σ) of the error were 3.5 and 5.9 mDa, respectively. It has been expected that the error in the m/z values of most of the fragment ions would be within ±10 mDa (2σ) (Table S1-I).

Table S1-I Comparison between the observed and theoretical m/z values of the fragment ions in the MS2T accessions of protonated molecules of kaempferol-3-glucoside-7-rhamnoside ([M + H]^+^, m/z 595.1).

| MS2T accession code | Keampferol aglycone  [C_15_H_11_O_6_]^+^  m/z 287.0556 (theoretical) | | Keampferol rhamnoside  [C_21_H_21_O_10_]^+^  m/z 433.1135 (theoretical) | |
| --- | --- | --- | --- | --- |
|  | Observed m/z (Da) | Δ (mDa) | Observed m/z (Da) | Δ (mDa) |
| ATH02p04021 | 287.0498 | –5.8 |  |  |
| ATH02p04223 | 287.0494 | –6.2 | 433.111 | –2.5 |
| ATH02p04231 | 287.0464 | –9.2 | n.d. |  |
| ATH02p04018 | 287.0568 | 1.2 | n.d. |  |
| ATH02p04027 | 287.0548 | –0.8 | 433.1104 | –3.1 |
| ATH02p04212 | 287.05 | –5.6 | 433.1047 | –8.8 |
| ATH02p04037 | 287.0661 | 10.5 | n.d. |  |
| ATH02p04042 | 287.051 | –4.6 | n.d. |  |
| ATH01p02729 | 287.0564 | 0.8 | n.d. |  |
| ATH01p02239 | 287.0561 | 0.5 | 433.1154 | 1.9 |
| ATH01p02735 | 287.0548 | –0.8 | 433.106 | –7.5 |
| ATH01p02242 | 287.065 | 9.4 | n.d. |  |
| ATH01p02726 | 287.0596 | 4 | 433.107 | –6.5 |
| ATH01p02235 | 287.0598 | 4.2 | 433.1137 | 0.2 |
|  | Average | –0.2 | Average | –3.8 |
|  | SD | 3.5 | SD | 5.9 |

n.d.: not determined.

*Distribution of number of fragment ions and intensity of base-peak ions in the MS2T library*

In order to evaluate the nature of the MS/MS spectral data in the MS2T libraries, the number of fragment ions and the intensity of the base peaks (the most abundant ion in the spectrum) in each MS2T accession were investigated (Table S1-II). The informative MS/MS data for structural estimation needs a spectrum containing more than two or three fragment ions with good signal-to-noise (S/N) ratio. In this regard, approximately 25% of the MS2T entries contain the MS/MS data with only one fragment ion or low S/N data whose intensity of the base-ion peak is less than 10 cps. These results indicate that the MS2T libraries contain many non-informative accessions. However, the MS2T accessions with low-quality MS/MS data are not likely to be tagged to the matrix since these were likely to be derived from the trace signals in the chromatograms. Indeed, the distribution of the number of fragment ions and intensity of the base-peak ions of the total 1543 accessions tagged to the matrix (Supplement data S3) shown in Table S1-III indicated that the ratio of the low-quality MS2T accessions decreased to 10.4% (160/1543); in other words, 90% of the MS2T data in the matrix were likely to be informative for the structural estimation of metabolites.

**Table S1-II** Distribution of the number of fragment ions and intensity of base-peak ions of the total 10194 accessions in two MS2T libraries (ATH01p and ATH02p).

| Number of fragment ions | Intensity of base-peak ions (cps) | | | | | | |  |  |
| --- | --- | --- | --- | --- | --- | --- | --- | --- | --- |
|  | <10 | 11–50 | 51–100 | 101–500 | 501–1000 | 1001–5000 | >500 | Total | (%) |
| 1 | 1124 | 511 | 35 | 6 | 1 | 0 | 0 | 1677 | 16.5 |
| 2 | 531 | 724 | 94 | 160 | 13 | 6 | 0 | 1528 | 15.0 |
| 3,4 | 348 | 1227 | 274 | 275 | 39 | 27 | 6 | 2196 | 21.5 |
| 5,6 | 84 | 732 | 261 | 236 | 43 | 33 | 8 | 1397 | 13.7 |
| 7,8 | 30 | 470 | 210 | 206 | 73 | 33 | 5 | 1027 | 10.1 |
| 9,10 | 7 | 219 | 155 | 147 | 35 | 19 | 3 | 585 | 5.7 |
| >10 | 6 | 427 | 453 | 742 | 89 | 59 | 8 | 1784 | 17.5 |
| Total | 2130 | 4310 | 1482 | 1772 | 293 | 177 | 30 | 10194 | 100.0 |
| (%) | 20.9 | 42.3 | 14.5 | 17.4 | 2.9 | 1.7 | 0.3 | 100.0 |  |

**Table S1-III** Distribution of the number of fragment ions and intensity of base-peak ions of the total 1543 accessions tagged to the matrix (Supplement data S2).

| Number of fragment ions | Intensity of base-peak ions (cps) | | | | | | |  |  |
| --- | --- | --- | --- | --- | --- | --- | --- | --- | --- |
|  | <10 | 11–50 | 51–100 | 101–500 | 501–1000 | 1001–5000 | >500 | Total | (%) |
| 1 | 49 | 28 | 3 | 0 | 0 | 0 | 0 | 80 | 5.2 |
| 2 | 38 | 62 | 4 | 9 | 2 | 1 | 0 | 116 | 7.5 |
| 3,4 | 30 | 157 | 37 | 51 | 16 | 8 | 4 | 303 | 19.7 |
| 5,6 | 7 | 112 | 38 | 42 | 21 | 17 | 5 | 242 | 15.7 |
| 7,8 | 4 | 70 | 41 | 51 | 10 | 8 | 4 | 188 | 12.2 |
| 9,10 | 1 | 32 | 33 | 40 | 9 | 5 | 0 | 120 | 7.8 |
| >10 | 0 | 72 | 124 | 231 | 38 | 26 | 0 | 491 | 31.9 |
| Total | 129 | 533 | 280 | 424 | 96 | 65 | 13 | 1543 | 100.0 |
| (%) | 8.4 | 34.6 | 18.2 | 27.5 | 6.2 | 4.2 | 0.8 | 100 |  |

*Difference between the total number of accessions in the MS2T library and number of MS2Ts tagged to the matrix.*

Although the MS2T libraries containing more than 10000 accessions were created in this study, approximately 500 peaks in the matrix were tagged by only 1543 MS2T accessions (Supplement data S2). This indicated that more than 8000 accessions in the MS2T libraries were not used for their original purpose. In order to investigate the reason for this gap, the accessions of the MS2T library were plotted by considering their retention times (x-axis) and the m/z value of the precursor ions (y-axis), as shown in Fig. S1-1. Although a total of 1543 accessions were tagged to the matrix (Supplement data S2), almost all the accessions were tagged to the peaks eluted from 1.0 to 6.0 min (Fig. S1-1A). On the other hand, the plot of the accessions that were not tagged to the data matrix revealed that the MS2T libraries contain a large number of accessions derived from artifact ions observed at m/z 100–200 and the hydrophobic compound eluted at the end of the chromatograms (Fig. S1-1B,C).

**A**

**B**

**C**

Figure S1-1 Retention time–mass number plot of the MS2T accessions on the MS2T data. Each accession of the MS2T library was plotted considering their retention times (x-axis) and m/z of precursor ions (y-axis). (A) A total of 1543 accessions that were tagged to the matrix (Supplement data S2). (B) A total of 8651 accessions that were not tagged to the matrix. (C) A total of 2750 accessions not tagged to the matrix with intense base-peak ion (above 50 cps).

*Technical problems in MS2T-based peak annotation*

In this study, MS2T data were acquired by performing a distinctive experiment from conventional metabolic profiling analysis. The creation of one MS2T library required many iterative analyses (16 h in total) in order to obtain as many MS2Ts as possible. However, once the MS2T libraries were created, the libraries could be applied to the annotation of metabolic profile data with similar metabolic profiles. It has been demonstrated that the dataset of *Ds*-transposon insertion lines (Fig. 7) was successfully tagged with MS2T libraries for tissue-specific data (ATH01 and 02) and could yield structural information for biological interpretation. However, the annotatability of the peaks in the matrix depends on the coverage of the MS2T assignment. In this study, only 50% of the peaks in the matrix can be tagged by the MS2T library. Moreover, it should be noted that the annotation of the newly observed peaks in the samples with different backgrounds requires performing an additional MS/MS analysis. Now, a more efficient method such as a nanoscale analysis with long gradient elution has been investigated for a more comprehensive acquisition of MS2T data.
