# Supplementary material for: MS/MS spectral tag-based annotation of non-targeted profile of plant secondary metabolites
Source: Plant J. 2008 Nov 11;57(3):555–77. doi: 10.1111/j.1365-313X.2008.03705.x (PMC2667644; doi:10.1111/j.1365-313X.2008.03705.x)
Supplement: Supplementary file 16 [file tpj0057-0555-SD16.doc]

**Supplemental text S2.** Detailed procedure for processing of metabolic profile data

*Generation of data matrix using MetAlign*

The profiling data files recorded in the MassLynx format (raw) were converted to the NetCDF format using the DataBridge function of MassLynx 4.1. From the set of NetCDF data files, the data matrix was generated using the MetAlign software (De V*os et a*l., 2007) (Fig. 1b step 2). The parameters used in data processing were as follows: Maximum amplitude: 10000; Peak slope factor: 1; Peak threshold factor: 6; Average peak width at half weight: 8; Scaling options: no; Maximum shift per scan: 35; Select min. nr. per peak set: 8 for the tissue specificity analysis data and 3 for the screen of *Ds*-inserted mutant lines.

By using this procedure, the data matrixes with unit mass data were generated, indicating that the high-resolution data acquired by the time-of-flight (TOF) analyzer have been discarded in this step. Although It has been pointed out that the accuracy of the *m*/*z* data obtained by the TOF analyzer (ca. 5–10 ppm) is insufficient for estimating the single candidate molecular formula (Kind and Fiehn, 2006), the high-resolution data have been considered as valuable information for estimating the molecular formula of the metabolites. Several peak-picking software packages have been developed for the high-resolution data and applied for the plant metabolomics studies (Katajamaa and Oresic, 2005).

On the other hand, an advantage of using the unit mass data is faster peak picking from the metabolic profiling data, which enables us to deal with a large-scale dataset in a high throughput analysis. In the case of the screening of *Ds*-inserted mutant experiment (Fig. 7), the generation of the matrix from the 219 raw data files was finished within one night by using a desktop PC (Pentium 4 3.0 GHz, 2 GB RAM). It should be noted that the high-resolution m/z data could be, at least in part, available from the precursor ion data of the corresponding MS2Ts. Further, the results of this study suggested that the unit mass data was sufficiently effective for the profiling of plant secondary metabolites. It is expected that the high-resolution data can be taken into consideration for more detailed metabolic profiling of large-scale datasets by improvements in the peak-picking software. The N toolbox described below can deal with high-resolution data with slight modification in the programs.

*Processing of data matrix*

The data matrix generated by the MetAlign were processed by the aid of in-house software written in Perl/Tk; the program was named N toolbox consisting of six programs, namely, Nprefilter, Nnormalizer, Nfilter, Nisotoperemover, aNannotator, and Nmotifsearch (Supplement data S4, Figs. S1-1, 3, 4, 5, and 6). The detailed methods for the processing of Arabidopsis tissue samples are described below.

*Pre-filtering* *(Nprefilter.pl)*

The peaks eluted before 0.85 min (scan number: 100) and after 14.0 min (scan number: 1650) were discarded using the Nprefilter.pl function (Fig. S1-1) to remove the low quantitative peaks eluted near the void volume, as well as the broad peaks eluted at the end of the chromatogram.


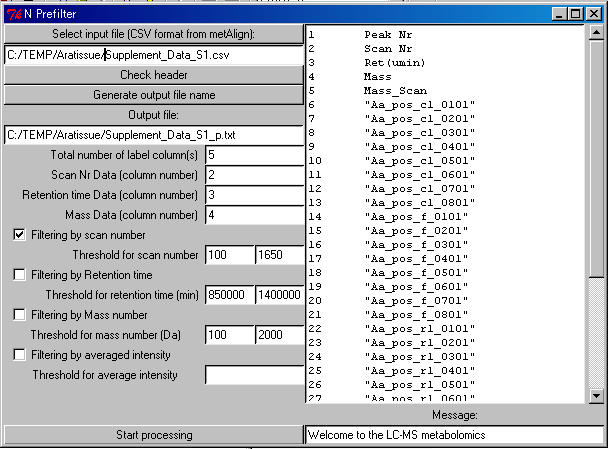


Figure S2-1 Screen shot of Nprefilter.pl.

*Data normalization (Nnormalizer.pl)*

The original peak intensity values were divided with that of the internal standards (lidocaine (m/z 235 [M + H]^+^, eluted at 4.19 min) and (–)-camphor-10-sulfonic acid (m/z 231 [M – H]^–^, eluted at 3.84 min) for the positive and negative ion modes, respectively) determined in the same samples to normalize the peak intensity values among the metabolic profile data. These standards were selected due to unnatural compounds that underwent stable ionization without being affected by the sample matrix (Fig. S1-2).

The purpose of the IS-based normalization was the correction of the errors other than those caused by ion suppression in contrast to the recently reported multi-internal standard approach for the correction of an ion-suppression effect in the focused metabolite analysis (Sysi-A*ho et a*l., 2007). The internal standards and LockSpray apparatus for the calibration of the mass-to-charge ratio (*m*/*z*) (Oika*wa et a*l., 2006; Suzu*ki et a*l., 2007; Wol*ff et a*l., 2001) were not employed since MetAlign only deals with unit mass data (De V*os et a*l., 2007), and it has been reported that the m/z accuracy (5–10 ppm) is insufficient for estimating a unique molecular formula (Kind and Fiehn, 2006).


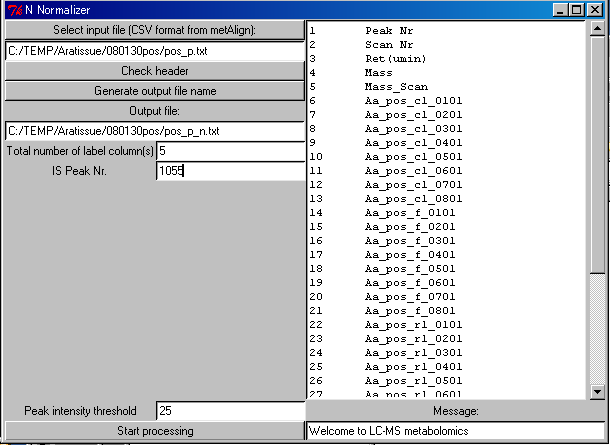


Figure S2-2 Screen shot of Nnormalizer.pl.

*Cutoff for low-intensity data (Nfilter.pl)*

Rows containing low-intensity and/or unreliable data were filtered by the following procedure using the Nfilter.pl function. In the case of the Arabidopsis tissue sample set, since each row included a total of 32 intensity data (4 groups × 8 replicates), rows including at least one group in which the intensity values of all the 8 replicates were above the cutoff value (0.0183, S/N = 5) were retained in the matrix, while the others were filtered (Fig. 1b step 4). The Nfilter.pl function was operated with the following parameters; Group: 8,8,8,8; Minimum number of samples in each group: 8,8,8,8; Peak intensity threshold: 0.0183 (Fig. S1-4).


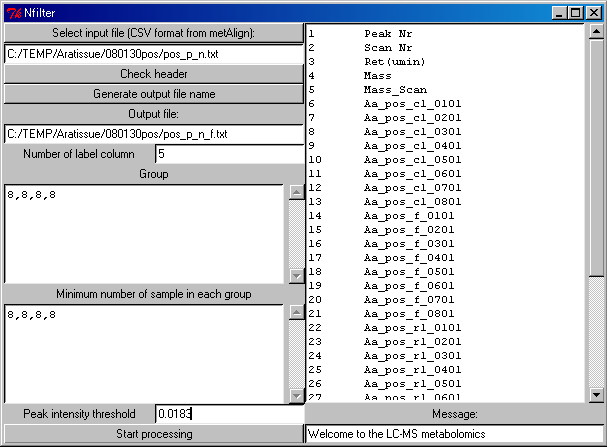


Figure S2-3 Screen shot of Nfilter.pl.

*Isotope peak deconvolution* (*Nisotoperemover.pl*)

It has been observed that several types of ions with different m/z values, such as fragment, adduct, and isotope ions, were generated in addition to the protonated molecule from a single metabolite during electrospray ionization (ESI). The deconvolution of the peaks is desired in order to reduce data redundancy, since the signals derived from these ions were recognized as distinctive peaks and recorded in different rows in the matrix. Among the redundant data in the matrix, the peaks derived from the isotope ions are easily detectable because these retention times and mass numbers are predictable from those of non-labeled signals. In addition, the intensities of the isotope peaks must be lower than those of the corresponding non-labeled signals, and the ratio of these intensities must be nearly identical among the samples. By using these characteristics, the peaks (rows) derived from the isotope ions in the matrix were eliminated using the following procedure in this study (Fig. S2-5).

(1) For a peak (row A), a candidate non-labeled peak (row B) with the highest correlation coefficient above the threshold value (r_thres_ > 0.8) was selected from the candidate peaks that were (i) eluted at similar retention times (within the retention time threshold (ΔR_t_ = 0.5 s)), (ii) observed at a smaller m/z value (within the m/z threshold (Δm/z < 3 Da)), and (iii) with a higher averaged intensity.

(2) If row B has another counterpart (row C), the counterpart of row A would be changed to that of row C.

(3) If row C has no counterpart, row A will be removed from the matrix, and its information will be described in the “deconvolution” column in row C.

The peak deconvolution method depends on the correlation coefficient of the intensity values among the rows in the matrix, implying that the method can deal with a matrix comprising more than 15–20 samples for calculating reliable correlation coefficient values.

Further, 20–30% of the rows derived from the isotope ions were discarded from the matrix in the fifth step (data not shown), suggesting that this step is important for reducing data redundancy. However, the resultant data matrix (Supplement data S2) still contains data derived from adduct and fragment ions. For the processing of the GC-MS data, efficient deconvolution software such as AMDIS have been developed using which a series of many fragment and their isotope peaks generated using electron-impact (EI) ionization can be deconvoluted based on the similarity between their shapes in the chromatograms (Broeckli*ng et a*l., 2006; Halk*et et a*l., 1999; Lis*ec et a*l., 2006). Although a few applications of the method for the treatment of the LC-MS data have been reported (Furtu*la et a*l., 2006; Roepenack-Laha*ye et a*l., 2004), most of the peak-picking software developed for the LC-MS data, such as MetAlign, have not included the deconvolution function because they employ distinct algorisms for peak picking and alignment of the profile data.

The abovementioned method has been applied to the deconvolution of adduct and fragment ions in the matrix by changing step (1) to the following:

(1) For a peak (row A), a candidate non-labeled peak (row B) with the highest correlation coefficient above the threshold value (r_thres_ > 0.8) was selected from the candidate peaks that were (i) eluted at similar retention times (within the retention time threshold (ΔR_t_ = 0.5 s)) and (ii) with a higher averaged intensity.

By using this method, it has been observed that two distinctive peaks of the eluted flavonol glucosides were deconvoluted into single metabolites since the retention time of these biosynthetic-related metabolites were essentially the same (data not shown). Thus, these methods were not employed for the deconvolution of the fragment and adduct peaks.


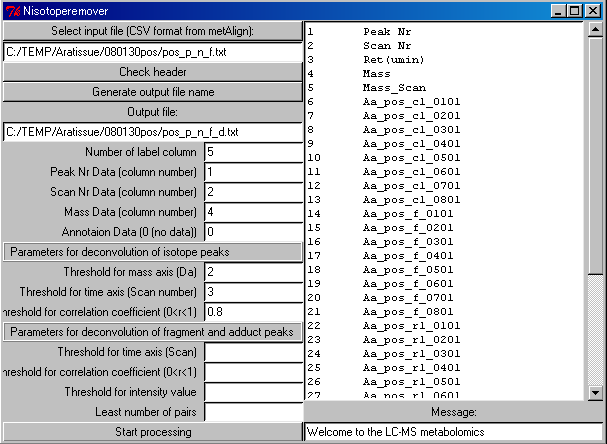


Figure S2-4 Screen shot of Nisotoperemover.pl.

*Peak annotation* (*Nannotator.pl*)

The retention time and m/z data of each row in the matrix were compared with those of all the accessions of the standard compound data, MS2T library, and curated annotation list (Supplement data S5, S1, and S6, respectively; Fig. S2-6). The accessions had identical m/z values and similar retention times (Δ_retention time_ < 0.05 min for standard compound and curated annotation, Δ_retention time_ < 0.15 min for MS2T library).


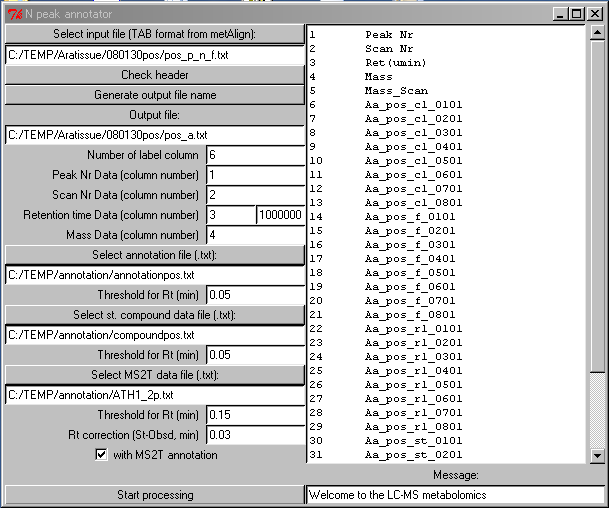


Figure S2-5 Screen shot of Nannotator.pl.

*Interpretation of MS2T data*

*KNApSAcK search*: High-resolution m/z data of the precursor ions of each MS2T were compared with those of the theoretical values of the protonated molecules [M + H]^+^ of the metabolites recorded in KNApSAcK (http://kanaya.aist-nara.ac.jp/KNApSAcK/), which contains the structural information of 21061 naturally occurring metabolites (Oika*wa et a*l., 2006). The error threshold in the m/z data was set to 5 mDa.

*MassBank search*: The MS/MS spectral data of the MS2Ts were queried using MassBank (http://www.massbank.jp/)—a database of high-resolution mass spectra of metabolites released by the JST-BIRD group (Taguc*hi et a*l., 2007)—by using the “Batch Search Service” function. A series of queried results were stored and used for the addition of structural information to peaks tagged with corresponding MS2Ts by evaluating the hits score (>0.8).

*Literature data search:* More than 900 MS/MS spectra of plant metabolites reported in the literature were collected and stored in a private database. The MS/MS spectral data of the MS2Ts were compared with these values according to their hit scores (>0.8) determined using the cosine product method (Stein and Scott, 1994) with the in-house Perl scripts. The details of the literature on the MS/MS spectra have been described elsewhere.

*Spectral motif search:* The motif search of the MS2T data was performed with in-house script written in Perl (Nmotifsearch).

*Validation of repeatability and intermediate precision of nontargeted metabolic profiling analysis using LC-MS*

The metabolic profiling analysis is a method used to determine the levels of metabolites in given samples. Although repeated analyses of the same sample are expected to produce identical results, the results inevitably contain errors due to various factors such as weighting and liquid handling in the extraction procedure, individual differences among the LC columns, and the operation conditions of the mass apparatus. Precision is an index of the error derived from the analytical method, and is determined as a standard deviation of the analysis. “Repeatability” (intra-assay precision) implies the precision within a single analysis using the same column, extraction buffer, and calibration of the mass spectrometer. “Intermediate precision” indicates the precision of the analyses performed under different conditions in terms of the analysis day, analyst, solvent, LC column, and calibration of the mass apparatus. The determination of these precisions for the analytical method is essential for evaluating the performance of the analysis. In this study, repeatability and intermediate precision were determined using the following method.

The homogenized frozen sample was prepared from bulk Arabidopsis shoot tissues. By using the method described in this report, triplicate analyses of the sample were repeated six times on different working days by changing the analytical column, extraction solvent, and mobile phase. A total of 18 samples were analyzed, and the data were processed using MetAlign to generate a data matrix. For each peak in the matrix, repeatability and intermediate precision were determined by the one-way analysis of variance as follows:

Data structure

|  | Repetition | | | | | | Total |
| --- | --- | --- | --- | --- | --- | --- | --- |
|  | 1 | 2 | 3 | 4 | 5 | 6 |  |
|  | x_1,1_ | x_2,1_ | x_3,1_ | x_4,1_ | x_5,1_ | x_6,1_ |  |
|  | x_1,2_ | x_2,2_ | x_3,2_ | x_4,2_ | x_5,2_ | x_6,2_ |  |
|  | x_1,3_ | x_2,3_ | x_3,3_ | x_4,3_ | x_5,3_ | x_6,3_ |  |
| Total | T_1_ | T_2_ | T_3_ | T_4_ | T_5_ | T_6_ | T_T_ |

Correction term: CT = T_T_^2^/(6 × 3)

Sum of squares:

S_T_ = ∑∑x_j,n_^2^ – CT

S_RW_ = ∑(T_j_^2^/N) – CT

S_r_ = S_T_ – S_RW_

Degrees of freedom:

Φ_T_ = JN – 1

Φ_RW_ = J – 1

Φ_r_ = J(N – 1)

Mean squares:

V_RW_ = S_RW_/Φ_RW_

V_r_ = S_r_/Φ_r_

Here, repeatability (s_r_) and intermediate precision (s_IM_) can be calculated using the following equations:

s_r_ = sqrt (V_r_)

s_IM_ = sqrt(s_r_^2^ + (V_RW_ – V_r_)/N)

The s_r_ and s_IM_ values were determined for 958 peaks in total in the matrix (Fig. S2-7). Although some peaks showed incorrect precision probably due to an unstable recognition of the peaks during the peak-picking process, the s_IM_ values of more than 95% of the peaks were below 10%. Based on this result, it is roughly expected that the metabolic profiling method can determine the levels of metabolites with a precision of 10%.

Figure S2-7. Precision of the metabolic profiling analysis. s_r_ (closed circles) and s_IM_ (open circles) values of 958 peaks in the matrix derived from the Arabidopsis shoot tissues are plotted. The x-axis represents the average intensity of each peak among the samples.

Literature Cited

**Broeckling, C.D., Reddy, I.R., Duran, A.L., Zhao, X. and Sumner, L.W.** (2006) MET-IDEA: data extraction tool for mass spectrometry-based metabolomics. *Anal. Chem.*, **78**, 4334-4341.

**De Vos, R.C., Moco, S., Lommen, A., Keurentjes, J.J., Bino, R.J. and Hall, R.D.** (2007) Untargeted large-scale plant metabolomics using liquid chromatography coupled to mass spectrometry. *Nature Protocols*, **2**, 778-791.

**Furtula, V., Derksen, G. and Colodey, A.** (2006) Application of automated mass spectrometry deconvolution and identification software for pesticide analysis in surface waters. *Journal of Environmental Science and Health. Part B*, **41**, 1259-1271.

**Halket, J.M., Przyborowska, A., Stein, S.E., Mallard, W.G., Down, S. and Chalmers, R.A.** (1999) Deconvolution gas chromatography/mass spectrometry of urinary organic acids—potential for pattern recognition and automated identification of metabolic disorders. *Rapid Commun. Mass Spectrom.*, **13**, 279-284.

**Katajamaa, M. and Oresic, M.** (2005) Processing methods for differential analysis of LC/MS profile data. *BMC Bioinformatics*, **6**, 179.

**Kind, T. and Fiehn, O.** (2006) Metabolomic database annotations via query of elemental compositions: Mass accuracy is insufficient even at less than 1 ppm. *Bmc Bioinformatics*, **7**, 234.

**Lisec, J., Schauer, N., Kopka, J., Willmitzer, L. and Fernie, A.R.** (2006) Gas chromatography mass spectrometry-based metabolite profiling in plants. *Nature Protocols*, **1**, 387-396.

**Oikawa, A., Nakamura, Y., Ogura, T., Kimura, A., Suzuki, H., Sakurai, N., Shinbo, Y., Shibata, D., Kanaya, S. and Ohta, D.** (2006) Clarification of pathway-specific inhibition by Fourier transform ion cyclotron resonance/mass spectrometry-based metabolic phenotyping studies. *Plant Physiol.*, **142**, 398-413.

**Roepenack-Lahaye, E.v., Degenkolb, T., Zerjeski, M., Franz, M., Udo Roth, Wessjohann, L., Schmidt, J.r., Scheel, D. and Clemens, S.** (2004) Profiling of Arabidopsis secondary metabolites by capillary liquid chromatography coupled to electrospray ionization quadrupole time-of-flight mass spectrometry. *Plant Physiol.*, **134**, 548-559.

**Stein, S.E. and Scott, D.R.** (1994) Optimization and Testing of Mass-Spectral Library Search Algorithms for Compound Identification. *J. Am. Soc. Mass Spectr.*, **5**, 859-866.

**Suzuki, H., Sasaki, R., Ogata, Y., Nakamura, Y., Sakurai, N., Kitajima, M., Takayama, H., Kanaya, S., Aoki, K., Shibata, D. and Saito, K.** (2007) Metabolic profiling of flavonoids in *Lotus japonicus* using liquid chromatography Fourier transform ion cyclotron resonance mass spectrometry. *Phytochemistry*, **69**, 99-111.

**Sysi-Aho, M., Katajamaa, M., Yetukuri, L. and Oresic, M.** (2007) Normalization method for metabolomics data using optimal selection of multiple internal standards. *BMC Bioinformatics*, **8**, 93.

**Taguchi, R., Nishijima, M. and Shimizu, T.** (2007) Basic analytical systems for lipidomics by mass spectrometry in Japan. *Methods in Enzymology*, **432**, 185-211.

**Wolff, J.C., Eckers, C., Sage, A.B., Giles, K. and Bateman, R.** (2001) Accurate mass liquid chromatography/mass spectrometry on quadrupole orthogonal acceleration time-of-flight mass analyzers using switching between separate sample and reference sprays. 2. Applications using the dual-electrospray ion source. *Anal. Chem.*, **73**, 2605-2612.
